# Mass azithromycin for prevention of child mortality among children with acute malnutrition: A subgroup analysis of a cluster randomized controlled trial

**DOI:** 10.1371/journal.pgph.0003875

**Published:** 2024-10-28

**Authors:** Ali Sié, Mamadou Ouattara, Mamadou Bountogo, Valentin Boudo, Thierry Ouedraogo, Clarisse Dah, Guillaume Compaoré, Elodie Lebas, Huiyu Hu, Travis C. Porco, Benjamin F. Arnold, Kieran S. O’Brien, Thomas M. Lietman, Catherine E. Oldenburg

**Affiliations:** 1 Centre de Recherche en Santé de Nouna, Nouna, Burkina Faso; 2 Francis I Proctor Foundation, University of California, San Francisco, San Francisco, California, United States of America; 3 Institute for Global Health Sciences, University of California, San Francisco, San Francisco, California, United States of America; 4 Department of Ophthalmology, University of California, San Francisco, San Francisco, California, United States of America; 5 Department of Epidemiology & Biostatistics, University of California, San Francisco, San Francisco, California, United States of America; University of Washington, UNITED STATES OF AMERICA

## Abstract

Children with acute malnutrition are at high risk of morality. Mass azithromycin distribution reduces all-cause mortality among children aged 1–59 months, and effects may be greater in underweight infants. Here, we evaluate the efficacy of azithromycin for reducing all-cause mortality in children aged 6–59 months with acute malnutrition (mid-upper arm circumference, MUAC, < 12.5 cm). Communities in Nouna District, Burkina Faso were 1:1 randomized to biannual mass distribution of single dose azithromycin or placebo to all children aged 1–59 months. Mortality was assessed during each census and treatment round. MUAC measurements were collected for all children. We evaluated the effect of azithromycin on mortality in subgroups of children aged 6–59 months defined by acute malnutrition (MUAC < 12.5 cm versus MUAC ≥ 12.5 cm). In children with MUAC < 12.5 cm, mortality rates were 51% lower among those living in azithromycin communities compared to placebo (incidence rate ratio 0.49, 95% confidence interval, CI, 0.25 to 0.99; incidence rate difference -18.1 deaths per 1,000 person-years, 95% CI -37.0 to -0.01), which was greater than the reduction in mortality among children with MUAC ≥ 12.5 cm (*P*-value for interaction on the relative scale = 0.09; *P*-value for interaction of the additive scale = 0.03). Children with acute malnutrition may benefit from single dose azithromycin above and beyond those without acute malnutrition.

**Trial registration:** ClinicalTrials.gov NCT03676764; https://clinicaltrials.gov/study/NCT03676764

## Introduction

In high mortality settings in sub-Saharan Africa, mass azithromycin distribution is being considered for preventing all-cause mortality [1]. The MORDOR study demonstrated a 14% reduction in all-cause childhood mortality with biannual mass azithromycin distribution. The largest effects were in Niger, with an approximately 18% reduction [2]. In MORDOR, the strongest effects were seen in infants <12 months of age, who had the highest mortality rates. This led to the hypothesis that azithromycin distributions targeted to children with the highest mortality rates may be the most effective [3]. Mass azithromycin distribution can select for antimicrobial resistance [4, 5]. Targeted distribution to those with the highest risk of mortality would reduce antibiotic consumption, which could minimize selection for antibiotic resistance.

Undernutrition is implicated in approximately 45% of childhood deaths in lower- and middle-income countries [6]. Antibiotics are routinely administered to children with severe acute malnutrition [7]. A subgroup analysis of infants enrolled in MORDOR demonstrated some evidence that infants with underweight (weight-for-age Z-score, WAZ, < -2) may benefit more from azithromycin compared to those who are not underweight [8]. Underweight is an indicator of both acute and chronic malnutrition and has been shown to be strongly correlated with mortality [9]. However, MORDOR was unable to assess undernutrition in older children.

The Child Health with Azithromycin Treatment (CHAT) trial was a cluster randomized trial in Nouna District, Burkina Faso that demonstrated an overall 18% reduction in mortality in communities receiving biannual azithromycin distribution to children 1–59 months compared to placebo [10]. While the effect sizes between CHAT and the Niger site of MORDOR were similar, mortality rates in CHAT were considerably lower (10 deaths per 1,000 person-years in the placebo arm in CHAT compared to 27.5 deaths per 1,000 person-years in the placebo arm of MORDOR). In CHAT, the largest effects on mortality were in children aged 24–59 months (27% reduction in mortality), in contrast to MORDOR where the largest effects were in infants. In CHAT, mid-upper arm circumference (MUAC) measurements were collected on all children, allowing for evaluation of effects of azithromycin on mortality in subgroups of children defined by acute malnutrition status. We hypothesized that azithromycin would be more effective in children with acute malnutrition compared to those without acute malnutrition.

## Methods

### Ethics statement

The trial was reviewed and approved by the Institutional Review Board at the University of California, San Francisco (Protocol #17–24230), and the Comité National d’Ethique pour la Recherche en Santé (Protocol 2018-8-111) and the Comité Technique d’Examine des Demandes d’Autorisation d’Essais Cliniques (Protocol 2018–134), both in Ouagadougou, Burkina Faso. The Institutional Review Boards approved all study activities, including MUAC screening and referral processes for acute malnutrition. Written informed consent was obtained from the caregiver of each participant.

### Trial methods

Complete methods and for the CHAT trial have been previously reported [11]. In brief, the CHAT trial was a 1:1 cluster randomized placebo-controlled trial evaluating the efficacy of biannual mass administration of a single, oral dose of azithromycin (20 mg/kg) compared to placebo for prevention of all-cause mortality among children aged 1–59 months. Communities were randomized to receive biannual mass azithromycin distribution to all eligible children in the community or a matching placebo. Communities were enrolled in the trial for 3 years (6 distributions of azithromycin or placebo) and vital status was assessed at each biannual census.

### Study setting

The trial took place in Nouna District, Burkina Faso. Nouna is located in northwestern Burkina Faso along the border with Mali. All communities in Nouna District were eligible except of Nouna Town, which is larger and more urban and overall has lower childhood mortality rates. Communities with populations larger than 2,000 participants were split into multiple clusters. Malaria transmission in the region is highly seasonal, with the high malaria transmission season occurring from approximately July through October. Seasonal malaria chemoprevention with sulfadoxine-pyrimethamine and amodiaquine is distributed monthly (July through October) during the high malaria transmission season to all children aged 3 to 59 months.

### Communities and participants

Prior to the study, a census of all communities in the district was conducted. All communities with the exception of Nouna Town, the capital of Kossi Province, were eligible for inclusion in the study. Communities with a population of over 2,000 individuals were split into multiple randomization units. All children aged 1–59 months in participating study communities were eligible for inclusion in the study if they weighed at least 3800 g, had no known allergies to macrolides, and had appropriate consent from their caregiver. Eligibility was assessed during each biannual census round, and children up to 65 months were censused to assess vital status but not offered treatment if they were ≥60 months.

### Intervention and randomization

Communities were randomized in a 1:1 fashion to biannual mass distribution with azithromycin to eligible children aged 1–59 months or a matching placebo. Randomization was stratified by whether the communities fell within an existing Health and Demographic Surveillance Site [12]. The placebo was identical in appearance, smell, and taste to the azithromycin, with the only difference being the active ingredient. Communities received biannual azithromycin or placebo distribution for 36 months, or 6 distributions in total, from August 2019 through February 2023.

### Anthropometric measurements

MUAC measurements were collected from all children at each biannual census round. MUAC was measured using a standard MUAC tape by the study staff member who was administering the study treatment. Study staff were trained in measurement of MUAC prior to each biannual census round [13–15]. Subgroups based on acute malnutrition for children aged 6–59 months were defined based on standard MUAC cutoffs for acute malnutrition: global acute malnutrition (MUAC < 12.5 cm), which includes both moderate and severe acute malnutrition, and severe acute malnutrition only (MUAC < 11.5 cm). Per Burkinabè national guidelines, children with MUAC < 11.5 cm (corresponding to severe acute malnutrition) were referred to government-run nutritional programs for diagnosis and treatment. Treatment of severe acute malnutrition includes provision of ready-to-use therapeutic food, Vitamin A, and antimicrobials including antibiotics, antiparasitics, and antimalarials if positive for malaria by rapid diagnostic test. Care for children with severe acute malnutrition was provided free of charge as part of the government-run program. The study’s mobile data application was programmed to alert the data collector to refer the child for treatment for any measurement of <11.5 cm.

### Primary outcome

The primary outcome for the trial was all-cause mortality as measured during the biannual census. Vital status was assessed for all children in the household who were eligible for treatment at the previous census round (up to 65 months of age). A child’s vital status was recorded as alive, died, moved, or unknown. Person-time was calculated by summing the person-time for each intercensal period. Children who were alive during both census rounds contributed the full person-time between the censuses. Children who died were assigned half the person-time in the census period. Children who moved or had an unknown vital status did not contribute person-time.

### Sample size

The sample size for the trial was based on the parent trial’s primary outcome, all-cause childhood mortality among children aged 1–59 months. During the census conducted prior to the start of the study, 341 trial clusters were identified, and were used for sample size calculations. We assumed an average community size of 1,000 people, and that 16.7% of the population would be in the 1-to-59-month age range. A base rate of 20 deaths per 1,000 person-years was assumed based on Institute for Health Metrics and Evaluation estimates [13]. A coefficient of variation of 0.34 was assumed based on the Niger site of the MORDOR trial, and 10% loss to follow-up. We estimated that 341 clusters would provide approximately 80% power to detect a 13.7% relative reduction in mortality.

### Statistical analysis

Because MUAC cutoffs for acute malnutrition are only used in children aged 6–59 months, analyses for subgroups defined by MUAC were restricted to children aged 6–59 months at the time of the MUAC measurement. The number of children with MUAC < 11.5 cm was too small for meaningful analysis, and all MUAC-based subgroup analyses were in children with global acute malnutrition (MUAC < 12.5 cm) or no acute malnutrition (MUAC ≥ 12.5 cm).

The analysis followed a similar strategy as the primary analysis [10]. Stratum-specific incidence rates, incidence rate ratios and incidence rate differences were estimated non-parametrically, with 95% confidence intervals estimated using a bootstrap that resampled communities with replacement (10,000 iterations). Tests of effect modification on the additive and multiplicative scales were based on a Wald test statistic from pooled logistic regression models of child-interval level data with either linear (additive) or complementary log-log (multiplicative) link functions, and robust standard errors clustered at the community level [14]. Because MUAC increases as children age, we assessed effect modification of the effect of azithromycin on mortality by age in months (as a continuous variable) in subgroups defined by MUAC < 12.5 cm and MUAC ≥ 12.5 cm using similar methods. All analyses were conducted in R version 4.2.1 (The R Foundation for Statistical Computing, Vienna, Austria). A two-sided P-value of <0.05 was considered statistically significant.

## Results

**Fig 1:**
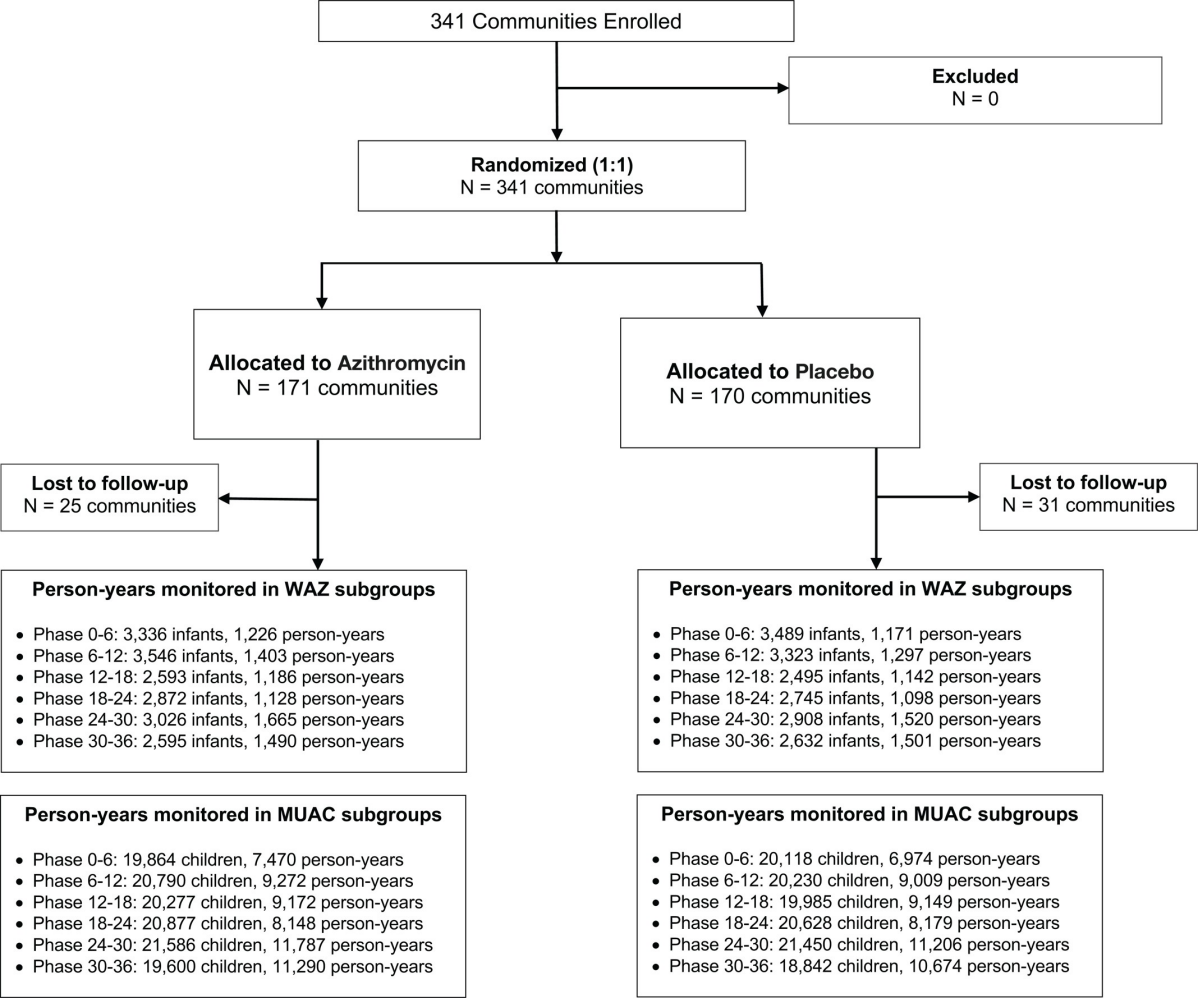
Flow diagram of study participants.

**Fig 2:**
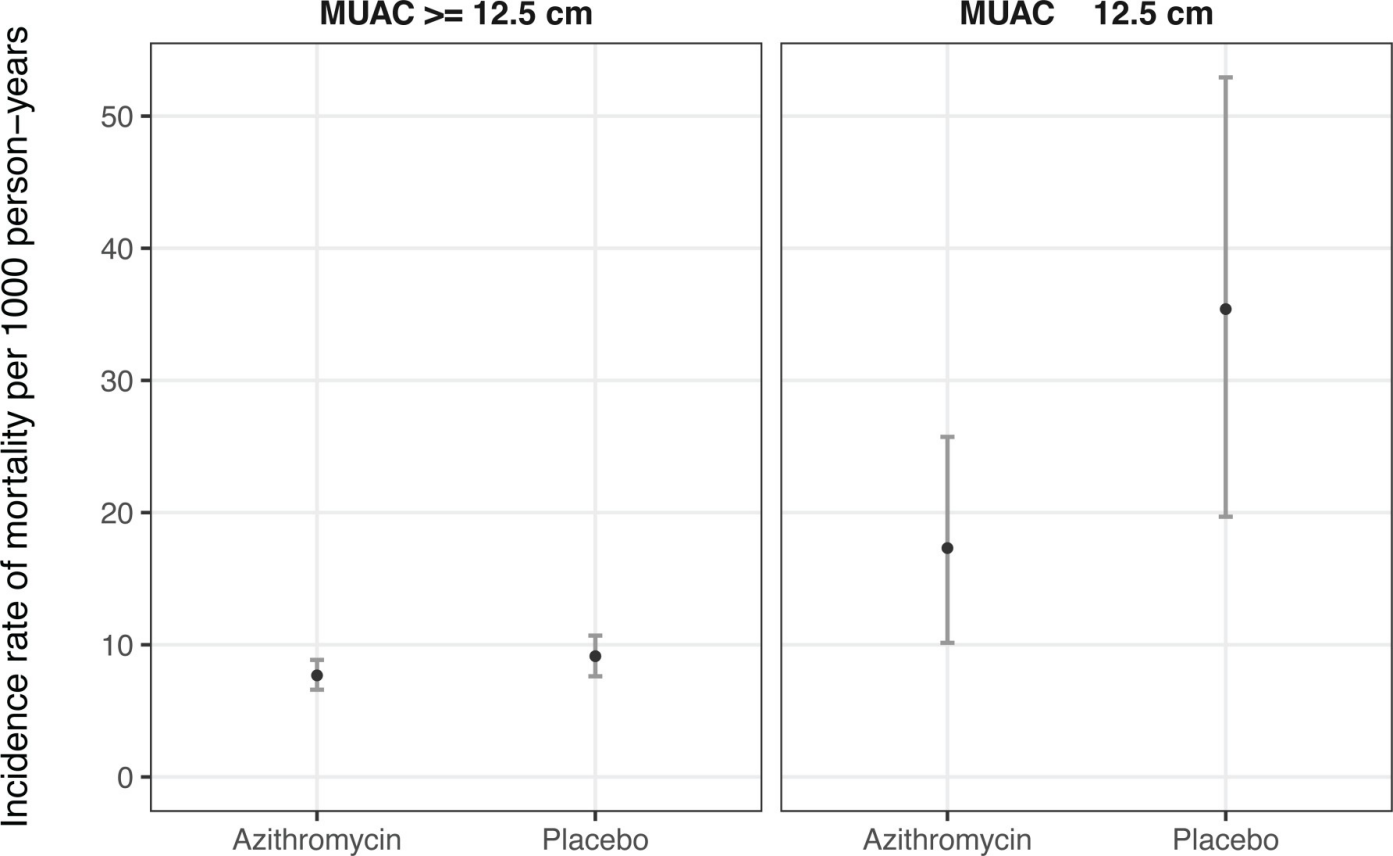
Incidence rates for mortality (per 1,000 person-years) among children aged 6–59 months with global acute malnutrition (MUAC < 12.5 cm) or no acute malnutrition (MUAC ≥ 12.5 cm) by randomized treatment assignment (azithromycin or placebo). Points indicate point estimates for the mortality rate and bars indicate 95% confidence intervals.

Of 341 clusters randomized, 297 contributed person-time to the analyses from August 2019 until February 2023, of which 145 were randomized to azithromycin and 152 were randomized to placebo (**Fig 1**). Clusters that were randomized but did not contribute person-time were not able to be censused or included due to security concerns in the study region, a possibility that was anticipated in the study’s statistical analysis plan. Approximately 50% of children in the study were female and median age among children contributing to the MUAC-based subgroups was 28 months (**Table 1**). A total of 1086 deaths were observed over 119,139 person-years in the study (overall incidence rate 9.1 deaths per 1,000 person-years, 95% CI 8.6 to 9.7). The incidence rate of mortality was 26.3 deaths per 1,000 person-years (95% CI 17.3 to 36.2) among children aged 6 to 59 months with MUAC < 12.5 cm, compared to 8.4 deaths per 1,000 person-years (95% CI 7.5 to 9.4) among those with MUAC ≥ 12.5 cm. Incidence rates in each subgroup by randomized treatment assignment are presented in **Fig 2**. As expected, mortality rates were higher in the subgroup of children with acute malnutrition regardless of whether they were receiving azithromycin or placebo.

**Table 1:** Baseline demographic characteristics among children 6–59 months participating in the CHAT cluster randomized trial.

	Age 6–59 months	
	Azithromycin	Placebo
N	32,667	33,319
Female sex, N (%)	16,243 (49.7%)	16,571 (49.9%)
Age in months, median (IQR)	28 (6 to 59)	28 (6 to 59)
MUAC < 12.5 cm	1,020 (3.1%)	1,107 (3.3%)

Abbreviations: IQR, interquartile range; MUAC, mid-upper arm circumference

**Fig 3:**
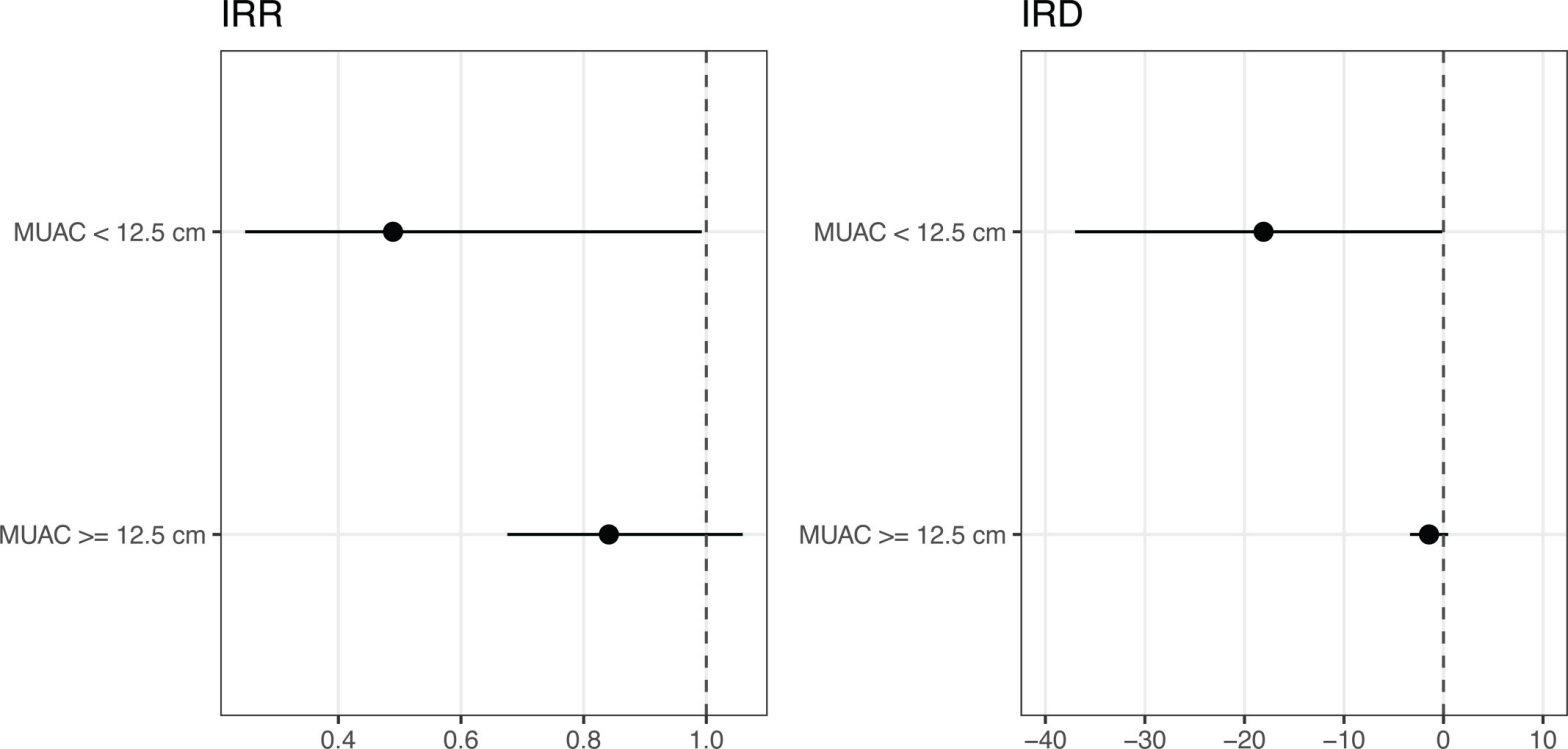
Incidence rate ratios (IRR) and incidence rate differences (IRD) for A) children aged 6–59 months receiving azithromycin vs placebo with MUAC < 12.5 cm (acute malnutrition) versus MUAC ≥ 12.5 cm (no acute malnutrition), and B) infants aged 1–11 months receiving azithromycin vs placebo with WAZ < -2 (underweight) versus WAZ ≥ -2 (no underweight). IRR estimates are log transformed. IRDs are the difference in number of deaths per 1,000 person-years. The dotted line indicates the null value, with estimate to the left favoring azithromycin, and estimates to the right favoring placebo.

Among children with acute malnutrition, there was a 51% relative reduction in the incidence rate of mortality among those receiving azithromycin compared to placebo (incidence rate ratio, IRR, 0.49, 95% CI 0.25 to 0.99). The effect of azithromycin for prevention of mortality was closer to the null for children without acute malnutrition, with an estimated 16% relative reduction in mortality among children without acute malnutrition receiving azithromycin (IRR 0.84, 95% CI 0.68 to 1.06), however there was no statistically significant evidence of interaction on the multiplicative scale (*P* = 0.09; **Table 2 and Fig 3**). In children with acute malnutrition, azithromycin led to 18 fewer deaths per 1,000 person-years compared to placebo (incidence rate difference, IRD, -18.1 per 1,000 person-years, 95% CI -37.0 to -0.1) compared to 1.5 fewer deaths per 1,000 person-years among those without acute malnutrition receiving azithromycin compared to placebo (IRD -1.45 deaths per 1,000 person-years, 95% CI -3.4 to 0.5, *P* for interaction on the additive scale = 0.03). We found no evidence of effect modification by age on the effect of azithromycin on mortality in children with acute malnutrition (*P* = 0.33) or without acute malnutrition (*P* = 0.90).

**Table 2:** Incidence rates, incidence rate ratios, and incidence rate differences among children aged 6 to 59 months by mid-upper arm circumference (MUAC) subgroup living in azithromycin compared to placebo communities.

	Azithromycin			Placebo				
	Person-Years	Deaths	Mortality Rate (Deaths per 1000 person-years)	Person-Years	Deaths	Mortality Rate (Deaths per 1000 person-years)	IRR (95% CI)	IRD (95% CI)
**Children aged 6–59 months**								
MUAC ≥ 12.5 cm	56,215	432	7.7	54,287	496	9.1	0.84 (0.68 to 1.06)^1^	-1.5 (-3.4 to 0.5)^2^
MUAC < 12.5 cm	942	16	17.3	904	32	35.4	0.49 (0.25 to 0.99)^1^	-18.1 (-37.0 to -0.01)^2^

Abbreviations: IRR, incidence rate ratio; IRD, incidence rate difference; MUAC, mid-upper arm circumference

^1^P-value for interaction on the relative scale = 0.09

^2^P-value for interaction on the additive scale = 0.03

## Discussion

In this subgroup analysis of a cluster randomized trial, children with acute malnutrition as measured by MUAC who received azithromycin had lower mortality compared to those receiving placebo, and the difference in mortality rates was greater than for children without acute malnutrition. These results are in line with previous work which has suggested that azithromycin may be most beneficial in subgroups of infants with underweight compared to those who are normal weight-for-age [8]. The present study included MUAC measurements for children aged 6–59 months, suggesting that effects previously seen in infants may be consistent across older children.

Antibiotics, most commonly amoxicillin, are routinely used as part of outpatient management for severe acute malnutrition (SAM) [7]. Antibiotics may increase weight gain in children with SAM [15, 16]. Children with SAM receiving antibiotics gained more weight in the weeks following admission to the nutritional program compared to those receiving antibiotics, but there were no differences 12 weeks after admission in Niger [17]. However, the evidence for any effect of antibiotics on mortality and nutritional recovery is less clear. A trial in Malawi suggested that antibiotics reduced mortality and improved nutritional recovery, and a trial in Niger was unable to demonstrate a benefit of amoxicillin for nutritional recovery and was underpowered for mortality, although there was a reduction in transfer to inpatient care among children receiving amoxicillin [15, 16, 18]. Children with global acute malnutrition (MUAC < 12.5 cm) have increased mortality rates compared to children without acute malnutrition. In the present study, the mortality rate in children with acute malnutrition in the placebo arm was approximately 3 times the mortality rate of children with MUAC ≥ 12.5 cm in the placebo arm. The current WHO good practice statement for children with moderate acute malnutrition indicates that these children should have access to a nutrient-dense diet to meet needs of recovery with consideration of provision of specially formulated foods under certain circumstances, but do not specify provision of anti-infectives, including antibiotics. At the time of the trial, the only intervention available for children with moderate acute malnutrition was nutritional counseling, and only children with severe acute malnutrition (MUAC < 11.5 cm) were referred to nutritional programs. The results of this study are hypothesis generating that mass drug administration with azithromycin may be beneficial for preventing mortality in children with acute malnutrition [2, 8]. As azithromycin can be provided as a single dose, it may be a logistically feasible intervention to consider for prevention of mortality among children with acute malnutrition in conjunction with development and scale-up of food-based interventions that can be delivered in the community.

Following the results of the MORDOR study [2], the World Health Organization (WHO) issued conditional guidelines regarding azithromycin distribution for childhood mortality [1]. MORDOR was unable to demonstrate evidence of an effect in lower mortality countries including Malawi and Tanzania [19]. As a result, the WHO guidelines focus on administration to 1–11 month-olds, who have the highest mortality rates, in settings where infant mortality is >60 per 1000 live births or child mortality is >80 per 1000 live births. Although overall mortality was lower in the present study than meets WHO guidelines for consideration of implementation of azithromycin, the CHAT trial found an 18% reduction in childhood mortality in communities that received mass azithromycin distribution [10]. As expected, the mortality rates were much higher in subgroups of children aged 6–59 months with acute malnutrition as defined by low MUAC. In lower mortality settings, a strategy targeting azithromycin to the highest mortality subgroups may be beneficial. However, despite the overall higher mortality in subgroups of children with undernutrition, the majority of deaths were in children without undernutrition due to the larger size of those groups. Thus, mass distribution strategies to all children regardless of undernutrition status may be the most optimal for averting the highest number of deaths [20]. Studies designed specifically to evaluate targeting anthropometry-based subgroups would be required before recommendations could be made related to targeted distribution strategies.

There are several limitations to consider in the present analysis. While children with acute malnutrition as defined by MUAC appeared to benefit more from azithromycin compared to those without acute malnutrition, subgroup sizes were small resulting in wide confidence intervals and uncertainty in estimates. These results can only be interpreted as hypothesis-generating. We cannot comment on whether a targeted-only strategy would reduce mortality based on these results. In the present study, all children were treated in the community, and it is possible that the mechanism of action of azithromycin for reducing mortality relies on reducing transmission of pathogens. If a substantial proportion of children in a community are untreated, they could interact with and transmit pathogens to malnourished children, reducing the efficacy of any targeted azithromycin strategy. For example, individually and household randomized studies, where entire communities are not treated at the same time, have generally failed to find evidence of an effect of azithromycin on mortality, whereas mass azithromycin administration to all children has been shown to reduce mortality in several separate studies [2, 21, 22]. Height measurements were not collected in the present study, and thus it was not possible to evaluate effects in subgroups of children who were stunted or had low weight-for-height Z-scores, an indicator of wasting. In addition, we had no information on edematous malnutrition. Children with concomitant wasting and stunting as well as wasting and underweight are at higher risk of mortality [23–25], and evaluation of the effects of azithromycin in these subgroups may yield valuable information.

In this subgroup analysis of the CHAT cluster randomized trial, mortality rates were higher in children with acute malnutrition compared to those without acute malnutrition. Children with acute malnutrition tended to have greater reductions in risk of mortality with azithromycin compared to placebo versus those without acute malnutrition, but most differences were not statistically significant. In lower mortality settings where widespread distribution may not be indicated, evaluation of targeting azithromycin distributions to subgroups of children with acute malnutrition may be warranted.

## Supporting information

S1 Protocol Manual of operations and procedures for the Child Health with Azithromycin Trial. (DOC)

S1 File Statistical analysis plan for the Child Health with Azithromycin Trial. (PDF)

S1 Checklist CONSORT 2010 checklist of information to include when reporting a randomised trial*. (DOC)
