# Supplementary material for: Mass azithromycin for prevention of child mortality among children with acute malnutrition: A subgroup analysis of a cluster randomized controlled trial
Source: PLOS Glob Public Health. 2024 Oct 28;4(10):e0003875. doi: 10.1371/journal.pgph.0003875 (PMC11515952; doi:10.1371/journal.pgph.0003875)
Supplement: S1 Protocol — (DOC) [file pgph.0003875.s001.doc]

Community Health Azithromycin Trial

Burkina Faso

Manual of Operations and Procedures

**Centre de Recherche en Santé de Nouna**

**The Francis I. Proctor Foundation, University of California, San Francisco**

**Catherine Oldenburg, ScD MPH**

**Ali Sié, MD PhD**

**Thomas Lietman, MD**

Mamadou Bountogo, MD

Boubacar Coulibaly, PhD

Cheikh Bagagnan, MS

Guillaume Compaoré, MD

Alphonse Zakane, MS

Mamadou Ouattara, MD

Valentin Boudo

Till Bärnighausen, MD ScD

Thuy D Doan, MD, PhD

Jeremy Keenan, MD MPH

Benjamin Arnold, PhD

Travis Porco, PhD MPH

Kieran O’Brien, MPH

Elodie Lebas, RN

Jessica Brogdon, MPH

Catherine Cook, MPH

Ariana Austin, MS

William Godwin, MPH

Ying Lin, MSPH

Fanice Nyatigo, BS

Huiyu Hu, MS

**Table of Contents**

[Abbreviations 5](#__RefHeading___Toc29803974)

[Chapter 1: Overview 6](#__RefHeading___Toc29803975)

[1.1. Executive Summary 6](#__RefHeading___Toc29803976)

[1.2. Objectives 6](#__RefHeading___Toc29803977)

[1.3. Study Partners 6](#__RefHeading___Toc29803978)

[1.4. Study Site 7](#__RefHeading___Toc29803979)

[Chapter 2: Context 9](#__RefHeading___Toc29803980)

[Chapter 3: Study Design 11](#__RefHeading___Toc29803981)

[3.1. Randomization 11](#__RefHeading___Toc29803982)

[Chapter 4: Study Eligibility 13](#__RefHeading___Toc29803983)

[4.1. Eligible Communities 13](#__RefHeading___Toc29803984)

[Eligible Individuals 14](#__RefHeading___Toc29803985)

[4.2. Study Schedule 15](#__RefHeading___Toc29803986)

[Chapter 5: Core and Non-Core Study Elements 16](#__RefHeading___Toc29803987)

[5.1. Mortality Study – All Communities 16](#__RefHeading___Toc29803988)

[**5.1.1.** **Core Elements** 16](#__RefHeading___Toc29803989)

[5.2. Mortality Plus Communities 18](#__RefHeading___Toc29803990)

[**5.2.1.** **Core Elements** 18](#__RefHeading___Toc29803991)

[**5.2.2.** **Non-core Study Elements** 18](#__RefHeading___Toc29803992)

[5.3 Individually randomized targeted treatment 19](#__RefHeading___Toc29803993)

[Chapter 6: Census 20](#__RefHeading___Toc29803994)

[6.1. Census 20](#__RefHeading___Toc29803995)

[6.2. Random Census Verification 23](#__RefHeading___Toc29803996)

[**6.3.** **Verbal Autopsy** 23](#__RefHeading___Toc29803997)

[**6.4.** **Validating the mortality outcome** 24](#__RefHeading___Toc29803998)

[Chapter 7: Registering Participants for Specimen Collection 25](#__RefHeading___Toc29803999)

[Chapter 8: Blood samples 25](#__RefHeading___Toc29804000)

[**8.1.** **Fingerstick** 26](#__RefHeading___Toc29804001)

[**8.2.** **Dried Blood Spots for Serology** 27](#__RefHeading___Toc29804002)

[Set-up drying area for TropBio bloodspots 29](#__RefHeading___Toc29804003)

[**8.3.** **Hemoglobin Test** 29](#__RefHeading___Toc29804004)

[**8.4.** **Thick and Thin Smears for Malaria** 30](#__RefHeading___Toc29804005)

[8.5. Materials for Blood Collection 31](#__RefHeading___Toc29804006)

[Chapter 9: Specimen Collection for Resistance Testing 32](#__RefHeading___Toc29804007)

[9.1. Population 32](#__RefHeading___Toc29804008)

[9.2 Nasopharyngeal Swabs 33](#__RefHeading___Toc29804009)

[**9.2.1** **Materials for Swab Collection for Resistance Testing** 33](#__RefHeading___Toc29804010)

[**9.2.2** **Protocol for Tubing and Handling of Samples** 34](#__RefHeading___Toc29804011)

[9.3 Stool Samples 35](#__RefHeading___Toc29804012)

[**9.3.1** **Stool Specimen Collection** 35](#__RefHeading___Toc29804013)

[**9.3.2** **Materials for Stool Specimen Collection** 37](#__RefHeading___Toc29804014)

[**9.3.3 Protocol for Fresh Frozen Stool Collection** 38](#__RefHeading___Toc29804015)

[9.4 Quality Control Measures for Specimen Collection 39](#__RefHeading___Toc29804016)

[Chapter 10: Training 40](#__RefHeading___Toc29804017)

[10.1 Standardization 40](#__RefHeading___Toc29804018)

[Chapter 11: Sample Organization, Transport, and Storage 40](#__RefHeading___Toc29804019)

[11.1 De-identification 40](#__RefHeading___Toc29804020)

[**11.2** **Specimen Transport** 40](#__RefHeading___Toc29804021)

[**11.3** **Specimen Storage** 41](#__RefHeading___Toc29804022)

[**11.3.1** **Short-term Sample Storage** 41](#__RefHeading___Toc29804023)

[**11.3.2** **Long-term Sample Storage** 41](#__RefHeading___Toc29804024)

[**11.4** **Catalog Specimens** 42](#__RefHeading___Toc29804025)

[Chapter 12: Study Medication 42](#__RefHeading___Toc29804026)

[12.1 Study Medication Description (from Pfizer, Inc.) 42](#__RefHeading___Toc29804027)

[12.2 Dosage Information 42](#__RefHeading___Toc29804028)

[12.3 Medication Procurement/Donation 43](#__RefHeading___Toc29804029)

[12.4 Medication Quality Control 43](#__RefHeading___Toc29804030)

[12.5 Antibiotic Distribution & Monitoring Coverage 43](#__RefHeading___Toc29804031)

[12.6 Adverse Reactions/Side Effects 44](#__RefHeading___Toc29804032)

[12.7 Adverse Events Systems 44](#__RefHeading___Toc29804033)

[**12.7.1** **Passive Adverse Events Monitoring** 44](#__RefHeading___Toc29804034)

[**12.7.2** **Active Adverse Events Monitoring** 44](#__RefHeading___Toc29804035)

[**12.7.3** **Adverse Events Data** 46](#__RefHeading___Toc29804036)

[12.8 Supply issues 46](#__RefHeading___Toc29804037)

[Chapter 13: Protection of Human Subjects 48](#__RefHeading___Toc29804038)

[13.1 Institutional Review Board Approval 48](#__RefHeading___Toc29804039)

[13.2 Informed Consent 49](#__RefHeading___Toc29804040)

[13.3 Risks and Benefits of Study Procedures 49](#__RefHeading___Toc29804041)

[**13.3.1** **Verbal Autopsy** 49](#__RefHeading___Toc29804042)

[**13.3.2** **Swabbing Procedures** 49](#__RefHeading___Toc29804043)

[**13.3.3** **Stool Collection** 49](#__RefHeading___Toc29804044)

[**13.3.4** **Blood Testing** 50](#__RefHeading___Toc29804045)

[**13.3.5** **Anthropometric Measurements** 50](#__RefHeading___Toc29804046)

[Chapter 14: Study Monitoring 51](#__RefHeading___Toc29804047)

[Chapter 15: Data and Safety Monitoring Committee Charter 51](#__RefHeading___Toc29804048)

[15.1 Primary Responsibilities of the DSMC 51](#__RefHeading___Toc29804049)

[**15.2** **DSMC Membership** 52](#__RefHeading___Toc29804050)

[**15.3** **Conflicts of Interest** 52](#__RefHeading___Toc29804051)

[**15.4** **Timing and Purpose of the DSMC Meetings** 52](#__RefHeading___Toc29804052)

[15.5 Procedures to Ensure Confidentiality and Proper Communication 53](#__RefHeading___Toc29804053)

[15.6 Statistical Monitoring Guidelines 54](#__RefHeading___Toc29804054)

[15.7 DSMC Contact Information 55](#__RefHeading___Toc29804055)

[Chapter 16: Data Collection, Management, and Security 56](#__RefHeading___Toc29804056)

[16.1 Scope of Data 56](#__RefHeading___Toc29804057)

[**16.2** **Data Storage, Management, and Security** 56](#__RefHeading___Toc29804058)

[**16.3** **Data Monitoring and Cleaning** 57](#__RefHeading___Toc29804059)

[Appendix 58](#__RefHeading___Toc29804060)

1. Abbreviations

CRSN: Centre de Recherche en Santé de Nouna

DCC: Data Coordinating Center

GPS: global positioning system

IRB: Institutional Review Board

MUAC: mid-upper arm circumference

NP swabs: nasopharyngeal swabs

PCR: polymerase chain reaction

STGG: skim milk tryptone glucose glycerin media

UCSF: University of California San Francisco

WHO: World Health Organization

DHMT: District Health Management Team

# Chapter 1: Overview

## Executive Summary

An estimated 7.7 million pre-school aged children die each year, the majority from infectious diseases.[^1^](#_ENREF_1) Mass azithromycin distributions for trachoma may have the unintended benefit of reducing childhood mortality.^1^ We recently demonstrated the biannual mass azithromycin distribution significantly reduces all-cause child mortality in a cluster randomized trial (MORDOR I) conducted in three diverse regions of Sub-Saharan Africa.^2^

Our long-term goal is to more precisely define the role of mass azithromycin treatments as an intervention for reducing childhood morbidity and mortality. We propose a cluster randomized trial designed to repeat the original study to confirm the original results in a different geographic study with similarly high child mortality, and to better understand the mechanism behind any effect of azithromycin on child mortality.

## Objectives

1: Determine the efficacy of biannual mass azithromycin distribution versus placebo in children aged 1-59 months for reduction in all-cause mortality. *We hypothesize that biannual distribution of azithromycin will lead to significantly reduced all-cause mortality among children aged 1-59 months after 36 months of treatment*.

2: Determine the efficacy of targeted azithromycin distribution to infants during an early infant healthcare visit (approximately 5th through 12th week of life) on infant mortality. *We hypothesize that infants receiving a single dose of azithromycin during early post-neonatal infancy will have significantly lower all-cause mortality compared to infants receiving placebo.*

**3:** Determine the mechanism behind the effect of biannual mass azithromycin distribution for reduction in child mortality.

## Study Partners

This study was jointly designed and will be jointly implemented by partners at CRSN and UCSF. CRSN and UCSF partners contributed equally to the development of this protocol. Funding for the study is provided by the Bill and Melinda Gates Foundation.

## Study Site

The study will be conducted in the Nouna District in northwestern Burkina Faso. It is situated about 300 kilometres north-west of Ouagadougou, the capital of Burkina Faso.

Nouna Health District is one of the six districts of Boucle du Mouhoun Health Region and covers the geographical area of Kossi Province in the western part of the country. Nouna is the capital of Kossi province. The health district comprises of the town of Nouna with a total population of 29,297 inhabitants and a rural area of about 235,426 inhabitants.

The multicultural society consists of 15 ethnic groups whereof the major ones are the Mossi, Bwaba, Marka, Samo, Gourounsi and Peuhl ethnic groups. The main socioeconomic activity of the population in the district is farming, similar to the rest of the country. The only exceptions are the Peuhl who are semi-nomadic cattle herders and dairy producers.

The health infrastructure consists of one District Hospital in Nouna (Centre Medical avec Antenne chirurgicale, CMA), and over 34 dispensaries (Centre de Santé et de Promotion Sociale, CSPS). The District Hospital in Nouna covers a population of 300,360 inhabitants. The Health Centers each cover a total population between 2,195 and 34,581 inhabitants. Due to deficient road infrastructure only 69.13% of health facilities are accessible for the DHMT all over the year with a mean distance of 8.48 kms (Nouna District action plan 2012).

Targeted treatment will also be done in the district of K. Vigué in the Haut Bassins region and in the district of Banfora in the Cascades region..


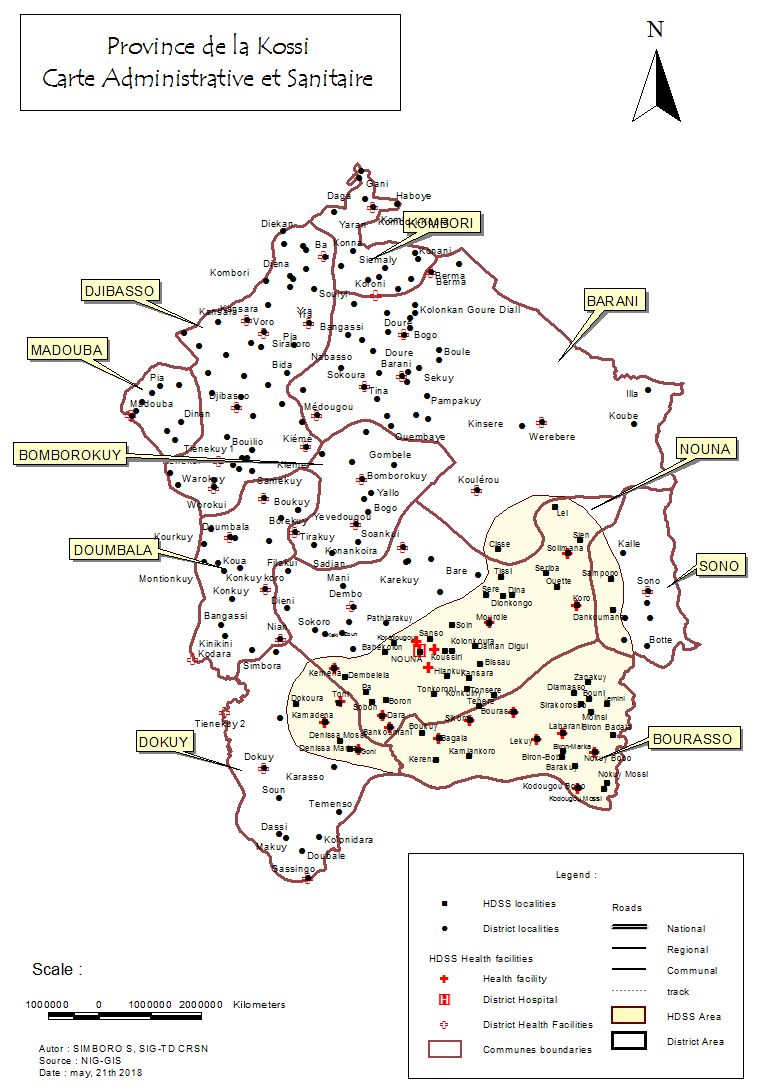


# Chapter 2: Context

Although child health and mortality are improving worldwide, children in the Sahel and sub-Sahel regions of West Africa have the greatest risks of mortality.^4,5^ Burkina Faso’s current under-5 mortality rate is estimated 110 per 1,000 live births^4^. Similar to other countries in the region, the major causes of child mortality in Burkina Faso are malaria, respiratory tract infection, and diarrhea. Malnutrition acts as a major underlying contributor to mortality.^6,7^ Interventions that address these underlying causes may be particularly efficacious for reducing mortality.

**Younger children are at a higher risk of mortality.** Approximately 2/3^rd^ of under-5 deaths occur during the first year of life.^4^ In general, the child mortality rate decreases as age increases. While some improvement has been observed, neonatal mortality is declining at a slower rate than post-neonatal childhood mortality.^4^ Many child health interventions are designed specifically for children over 6 months of age, such as vitamin A supplementation, seasonal malaria chemoprevention, and lipid-based nutritional supplementation. Identification of strategies that are safe and effective for the youngest children will be required to address persistently high rates of neonatal and infant mortality.

**The MORDOR I study demonstrated a significant reduction in all-cause child mortality following biannual mass azithromycin distribution.** Across three diverse geographic locations in sub-Saharan Africa (Malawi, Niger, and Tanzania), biannual mass azithromycin distribution over a two-year period led to a 14% decrease in all-cause child mortality. In Niger, 1 in 5-6 deaths were averted. These results are qualitatively similar to those of a previous study of mass azithromycin distribution for trachoma control in Ethiopia, which found reduced odds of all-cause mortality in children in communities receiving mass azithromycin compared to control communities.^1^

**In MORDOR I, the strongest effect of azithromycin was in the youngest cohort of children.** Across all three countries, the strongest effect of azithromycin was consistently in children 1-5 months of age, with an approximately 25% reduction in all-cause mortality. However, MORDOR I was not optimized to target the youngest age groups. Although children as young as 1 month were eligible, biannual distributions might not reach some children until 7 months of age. On average, children were first treated at 4 months. Given that there may be a substantial benefit to treating children at younger ages, azithromycin strategies that are designed to target younger age groups may be even more beneficial for reducing child mortality.

**Here, we propose a randomized controlled trial designed to evaluate the efficacy of mass and targeted azithromycin strategies for child mortality.** In the rural northwestern district of Nouna in Burkina Faso, we propose to randomize villages to biannual mass azithromycin distribution or placebo. This study was designed by CRSN and UCSF partners to confirm the results of MORDOR I, evaluate an alternative health systems distribution point (targeted treatment) for delivery of azithromycin to young children, and to provide a platform for evaluation of potential mechanisms behind the effect of azithromycin by collecting and processing additional specimens and tests.

# Chapter 3: Study Design

The research team will assess childhood mortality over three years, comparing communities where children aged 1-59 months receive biannual oral azithromycin and/or targeted azithromycin during the 5^th^-12^th^ week of life in conjunction with the first Expanded Programme on Immunization (EPI) vaccine visit, the BCG vaccine visit and the 42-day postnatal health visit/well-child visit or biannual placebo and targeted placebo. All eligible communities in Nouna District will be randomized (278 communities). A random sample of 48 (24/arm) communities from within the HDSS will be selected to participate in the “Mortality Plus” study, which will entail an annual morbidity exam among 15 randomly selected children per community to monitor infectious disease morbidity, nutritional status, and macrolide resistance. All communities will contribute to the mortality outcome. All biologic specimens collected as part of the morbidity and resistance outcomes will be stored and made available to other investigators for further laboratory testing at the conclusion of the study, per Gates Foundation guidelines.

## Randomization

**Randomization of Treatment Allocation.** All eligible communities in Nouna District will be randomized in a 1:1 fashion to biannual azithromycin or placebo. Targeted treatment will be randomized 1:1 individually to azithromycin or placebo in the nouna, K vigué and banfora districts. Refer to SAP for randomization details.

**Study Participants:** At months 0, 12, 24, and 36 a random sample of children will be selected using a computer-generated simple random sample for exams and sample collection to monitor for morbidity and resistance in the Mortality Plus communities.

**Figure 1: Trial Profile**


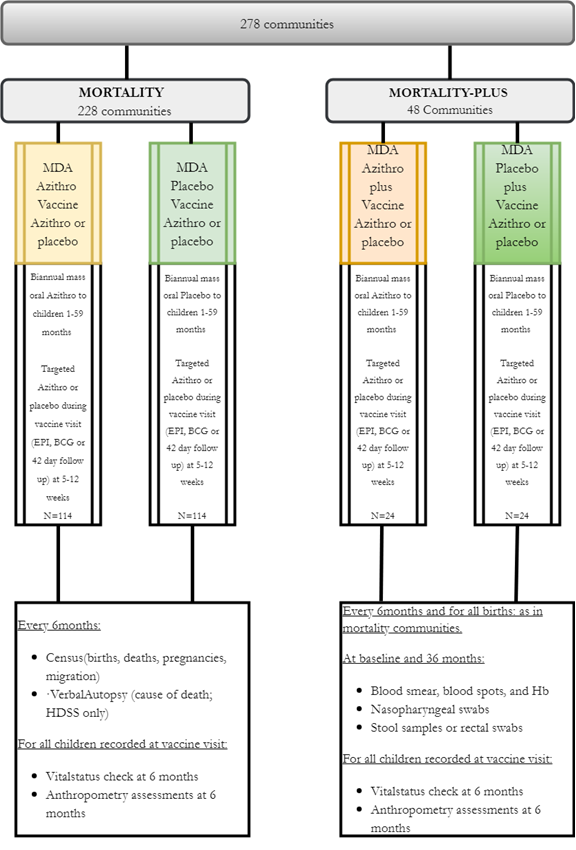


# Chapter 4: Study Eligibility

## Eligible Communities

To be eligible for the trial, a community must meet the following criteria:

1. The community location in target district.
2. The community leader consents to participation in the trial (this does not obviate the need for individual consent, but without overall leadership consent, the community as a whole cannot be part of the trial).
3. Eligible communities estimated population no more than 2,000 people. All communities with an estimated population of more than 2000 people will be split into 2 or more randomization units
4. The community is not in an urban area.

## Eligible Individuals

**Mortality Study:**

**Census:** The study can be thought of as consisting of seven 6-month segments, each of which starts with a census and ends with a follow-up census. All children in the study communities aged 1-59 months (up to but not including the 5^th^ birthday) at the initial census of each segment are eligible to participate in the subsequent 6-month segment of the study. Note that the information for children erroneously entered into the census (e.g., children younger than 1 month or ≥ 60 months) can be corrected at the subsequent treatment, subsequent census, or at a verbal autopsy later in the study. However, these changes will not be applied retroactively; these misclassified children will still be included in the study population for that 6-month segment and any deaths will be counted toward the primary outcome. In addition, all children listed on the initial census for that 6-month segment will be included in the outcome, regardless of whether they received the study drug.

**Treatment:** Individuals allergic to macrolides or azalides will not be given the study antibiotic azithromycin, but will be included in the outcome. Children weighing less than 3.8 kg will not be given treatment either.

**Birth Notification:** All births in all study communities will be recorded over the duration of the study.

**Mortality Plus Communities:**

**Census:** The criteria for being included in the census are the same as the Mortality Study, as described above.

**Treatment:** The inclusion and exclusion criteria for treatment are the same as for the Mortality Study, as described above.

**Examination & Sample Collection:** A random sample of children aged 1-59 months (up to but not including the 5^th^ birthday) are eligible for examination and sample collection. As described above, the random sample will be a simple random sample based on the previous census. These individuals will likewise be selected from the previous census.

## Study Schedule

The schedule for examination and treatment is shown below in Table 1:

|  | **MORTALITY** | **MORTALITY PLUS** |
| --- | --- | --- |
| **Ongoing** | Birth notification  1^st^ EPI or BCG or 42-day postnatal visit/well-child visit azithro or placebo  6 mo vital status  6 mo Anthropometry assessments | Birth notification  1^st^ EPI or BCG or 42-day post natal/well-child visit azithro or placebo  6 mo vital status  6 mo Anthropometry assessments |
| **MORDOR 0**  **Aug-19 to Jan-19** | Census  Azithro or placebo | Census  Swabs  Blood  Anthropometry  Azithro or placebo |
| **MORDOR 6**  **Feb-19 to Jul-20** | Census  Verbal Autopsy  Azithro or placebo | Census  Azithro or placebo |
| **MORDOR 12**  **Aug-20 to Jan-20** | Census  Verbal Autopsy  Azithro or placebo | Census  Azithro or placebo |
| **MORDOR 18**  **Feb-20 to Jul-21** | Census  Verbal Autopsy  Azithro or placebo | Census  Azithro or placebo |
| **MORDOR 24**  **Aug-21 to Jan-21** | Census  Verbal Autopsy  Azithro or placebo | Census  Azithro or placebo |
| **MORDOR 30**  **Feb-21 to Jul-22** | Census  Verbal Autopsy  Azithro or placebo | Census  Azithro or placebo |
| **MORDOR 36**  **Feb-22 to Jul-22** | Census  Verbal Autopsy  Azithromycin for all  Birth History (subset of communities | Census  Swabs  Blood  RDT  Anthropometry  Swab targeted treatment group |
| **Note: Only children aged 1 month to 59 months in each community will be treated during mass drug administration** | | |

MORDOR II Study Schedule

# Chapter 5: Core and Non-Core Study Elements

An overview of core and non-core elements for the mortality and morbidity study is provided here, but will be described in more detail in the following chapters.

## Mortality Study – All Communities

### **Core Elements**

We will conduct the following study activities for the mortality study:

**Training**Standardization activities before each biannual census will consist of didactic classroom instruction and mock census activities, followed by in-field training.

**Pre-census and mapping questionnaire**

Before the beginning of the study, we will perform a pre-census to be able to list all compounds and household in our study area. This pre-census will help us organize our data census collection by knowing the area we will be working on.

The pre-census will include all questions ask during regular census (see chapter census below) and will include a mapping questionnaire about the characteristics of each household:

- Wall, roof and ground of the house
- Type of water supply
- Latrinization
- Electricity
- Telephone (if exist take number with permission)
- Cooking
- Education of the head of household and mother/guardian
- Child individual information:
  - Pre schooling
  - Breastfeeding
  - Use of bednet
  - Handicap

**Census**An enumerated population census for 0 – 60 month olds (focusing on this age group) will be conducted every 6 months by trained field workers masked to study arm, recording births, deaths, and migration of children eligible for treatment. Pregnant women will be noted at each census, to maximize inclusion of newborns on the subsequent census.

**Core Census Elements**

The following elements will be considered part of the core HDSS census activities:

- Enumeration of the compound
- Full enumeration of household members with emphasis on children aged 0-60 months (age, sex) in a way that each child could be link to his mother
- Enumeration of pregnancies
- Recording of caregiver for each child <60 months
- Recording head of household
- Mid-upper arm circumference measurement for all children aged 0-60 months
- GPS coordinates

**Random Census Verification**A repeat census will be conducted in a random selection of households at each study visit. Personnel will be different from the original census. This will allow us to assess whether the census identified all births, deaths, and migratory episodes relative to the previous census.

**VerbalAutopsy**Verbal autopsy will be conducted in the district according to current methods for measuring verbal autopsy for all children aged 1-59 months who died during the study.^3^ The interview is conducted with the caregivers or relatives, using the WHO standard verbal autopsy questionnaire WHO-VA-2016. The interview usually takes place two months after the event with the person who assisted the deceased before the death. The data collected will be coded using InterVA 4.

**Treatment**Children aged 1-59 months on the current census will be offered weight- or height-based (<1 year and older children who can’t stand will be weighted; ≥1 year will be measured with a flexible dosing stick), directly observed, oral azithromycin suspension (or oral placebo) every 6 months for 3 years as performed in trachoma programs. Specifically, individuals are eligible on or after their one month birthday, and prior to the day of their fifth birthday.

At the final treatment distribution, CHAT 36, all children 1-59 months old will be given a single dose of azithromycin. Placebo will not be utilized at the final phase.

**Antibiotic Coverage Surveillance**We will estimate antibiotic coverage from the most recent biannual census records. At the end of each treatment round at months 6, 12, 18, 24, and 30 we will identify any children who have missed 2 or more consecutive treatments, and forward this information to the census team.

**Birth history at the 36 months visit**

At the final visit (36 month) we will obtain birth history in a subset of communities. All women in childbearing age living in selected communities will be asked if they experienced births in the last 10 years. Name of women, age, name of children and age or date of birth will be collected. Vital status of each birth will be recorded.

## Mortality Plus Communities

### **Core Elements**

All core elements described in 5.1.1 will also be conducted in the Mortality Plus communities.

The designation “core elements” means that all study communities will be performing the study activity.

In all study sites, we will perform the following tests on a random sample of 15 children aged 1-60 months from each community at baseline, 12, 24, and 36 months:

- Blood samples (dried blood spots) for malaria and anemia
- Nasopharyngeal swabs for pneumococcal macrolide resistance
- Stool samples or rectal swabs to assess for macrolide resistance
- Anthropometric assessments
- Rapid diagnostic test for malaria (at 36 months only)

Samples will be processed at the CRSN laboratory for microbiological culture, targeted PCR, serologic, thin and thick smears, and ova and parasite tests. Samples for microbiome analyses and specialized tests required by the Gates Foundation will be shipped to the United States.

At the final visit (36 months), in the mortality plus communities, we will obtain one rectal swab from up to 10 children per community who participated in the targeted treatment distribution.

### **Non-core Study Elements**

The designation “non-core” element means that not all study communities will be participating in the activity.

Mother MUAC assessment

In a subset of 20 villages, we will train mothers/guardians on measuring MUAC on their children weekly. We will train mothers/guardians to bring children to the nearest CSPS for evaluation if MUAC is <125mm.

**Passive Surveillance**

In each Centre de Santé et de Promotion Sociale (CSPS, community health facility), we will conduct morbidity passive surveillance. Each CSPS will be equipped with a tablet for electronic capture of health facility visits. Each visit will be recorded, including the reason for the visit (e.g., fever, diarrhea, malnutrition, etc), the village of residence, the person’s age and sex, diagnosis (e.g., malaria, pneumonia, etc), treatment (e.g., antibiotic, antimalarial, etc), and timing of the visit (e.g., first versus follow-up visit). Note that this data is already routinely collected on paper forms. Identifying information like names will not be collected.

In each CSPS included in the Mother MUAC assessment, we will collect data on malnutrition including name of participants in the program, study ID, village of residence, date of admission in the program, measurements of weight and height, treatment received and outcome. Notes that this data is already collected on paper forms

Passive surveillance in the district hospitals located in our study area: we will collect study ID, name of participant, village of residence, date of admission and discharge, reason for hospitalization, treatment, and outcome of hospitalization for all children hospitalized under 6 months of age. Notes that this data is already collected on paper forms

## 5.3 Individually randomized targeted treatment

**Recruitment**

There are three occasions in which children could be recruited for the targeted treatment:

1)During the 1st BCG visit at approximately 5 weeks of life if the child is at least 29 days old.
2) During the 6 week (42 days) postnatal follow-up visit or any other well-child visit happening during the age of 5 weeks to 12 weeks
3) When children are attending their first EPI vaccine visit at approximately 12 weeks of life (1st EPI vaccine visit).

**Enrollment**

The children will be enrolled for targeted treatment at the health center or during other health outreach in the community after obtaining written consent from at least one guardian. Enrollment will happen at the Nouna district, the K. Vigué district and Banfora districts

The children enrolled have to be living in a participating study community and be aged 28 days to 12 weeks.

**Anthropometric Baseline assessments**

All children enrolled in the study will undergo anthropometry: we will measure height, weight and middle arm circumference of each child.

**Treatment**

In all communities, children attending local health posts or children present at community health outreach will have the opportunity to receive a dose of azithromycin or placebo. Children will be individually randomized to receive placebo or azithromycin. Receipt of treatment will be recorded on the child’s study card. This treatment will occur whether the targeted treatment occurs during other health outreach or when the caregiver seeks vaccination at the health post or when the child visits the health post for a well-child visit. Community health workers will conduct a household visit when the mother and child do not come to the health post.

**2-week infant adverse event survey**

To identify any adverse events associated with the individually treated children, the research team will perform an adverse event survey approximatively 2 weeks after the treatment has been administered in a random subset of children. 10% of the children treated as part of the study will be randomly selected and the 2-week IAES will be performed. This survey will be performed by the census workers masked to treatment arm. A structured questionnaire will be performed to elicit adverse events following treatment, followed by an open-ended question. Specifically, we will ask the primary caregiver about the following symptoms during the time since the previous antibiotic distribution: abdominal pain, vomiting, diarrhea, constipation, hemorrhoids or rash.

**Six-month Mortality**

All children treated in all study communities will be followed for 6 months for vital status assessment. At approximately 6 months of age, a field worker will assess the vital status of the child (alive, died, unknown) and their current residence (residing in the household, moved, unknown). This visit will be an in-person visit to the health post.

**Six-month Anthropometry assessment**

All children treated during the targeted treatment visit in all study communities will be followed for anthropometry assessments at approximatively 6 months of age. The child will be measured, weighted and we will measure the middle upper arm circumference. This procedure will be done at the health post during the 6-month old vaccine visit.

**Rectal swab collection**

During the 36 months visit in the mortality plus communities we will obtain one rectal swab from up to 10 children per community who participated in the targeted treatment.

# Chapter 6: Census

## Census

**Census Team**

Census workers will be selected by the CRSN study coordinator. These individuals may have different qualifications and educational backgrounds, but, at a minimum, each census team member should be computer-literate, such that they are able to operate a tablet computer and type on its keyboard. In addition, several supervisors will be present for the duration of the census to monitor census workers.

**Census Training**

Census workers will be trained at the beginning of the study and refresher trainings will be offered as needed for the duration of the study. Training will start with reviewing the census data collection software on the tablet computer, care of the tablets, charging of the tablets, etc. The training will then proceed to a demonstration of the use of the software at a mock household, including common problems that staff may encounter (e.g., no one at home, GPS function not working, software crashing). In the final part of the training, the study coordinators and investigators will accompany team members to several communities and observe the census activities.

**Census Software**

The census will be directly entered into a tablet computer. The software will capture information about each child aged 0-5 in each household: name, age, sex, father’s name, and mother’s name, and will also register any pregnant women. The GPS coordinates will be documented for each household at the entrance to the household. At follow-up censuses, team members will identify each household on the existing census, and will update the status for each child:

- STATUS: Alive, slept in household last night
- STATUS: Alive, but not in household
  - ABSENCE: <1 month
    - Is he/she coming back within 1 week?
      - Yes (mop-up)
      - No
      - I don’t know
    - INFORMANT: household member, neighbor, village chief, other
  - ABSENCE: ≥ 1 month
    - MOVE: Moved within community
      - INFORMANT: household member, neighbor, village chief, other
    - MOVE: Moved outside of community
      - INFORMANT: household member, neighbor, village chief, other
- STATUS: Died
  - PLACE: Child living in community when died
    - INFORMANT: household member, neighbor, village chief, other
  - PLACE: Child had moved out of community when died
    - INFORMANT: household member, neighbor, village chief, other
- STATUS: Unknown
  - INFORMANT: household member, neighbor, village chief, other

Whenever a new individual is added to the census, the software will automatically assign each individual to a universal unique identification number as well as a study identification number.

**Census Data Uploading**

The census will be collected on tablet computers with 3G mobile and Wi-Fi capabilities. There will be 3 options for uploading data to the database. First, and most desirable, a SIM card with data plan can be purchased for each tablet, and the data uploaded via cell towers once per day (at the end of the day). This option is most desirable because it minimizes data loss in the case of a lost, stolen, or damaged device. In addition, this option will not require each tablet computer to be in contact with a Wi-Fi hub. As a second option for uploading, each census supervisor will have access to a Wi-Fi hub, and the supervisor can visit the census teams to upload data regularly. This option is less desirable, because the data will be uploaded less frequently. As a third option, the data can be uploaded at a central study site, either via Wi-Fi or micro USB cable directly into a computer. This option is least desirable because is not feasible to take the tablet computers to the central site regularly given the large geographical areas of the study.

**Census Supervision**

The CRSN study coordinator will supervise all census activities. Formal checks of census quality will be conducted through the random census verification. In addition, the study coordinator and CRSN GIS team will visualize all censused households using imagery from GoogleMaps. The goal of this activity will be to minimize the chances of missing large neighborhoods or specific regions within study communities. The study coordinator will also check the data entry progress for each community, paying special attention at the follow-up censuses, as to whether there are any missing data for the “vital status” variable (i.e., present, dead, absent). Once the study coordinator is confident that the entire community has been reached, and that the amount of missing data are acceptable (defined as <10% of children in a community), the study coordinator (or other research team member) will certify the census data collection for that community complete via Salesforce. Changes can be made to the record at different time points, but these changes will not be reflected until the subsequent census. All changes are time and date stamped in the database.

**Census Timing**

The census will be performed prior to each mass azithromycin/placebo distribution. Study sites may choose to perform the census activities over a discrete time period (e.g., all communities completed over a 1-month period, requiring census activities to take place simultaneously in many communities at once) or alternatively in a “rolling” fashion (e.g., all communities completed over a 6-month period, requiring fewer census teams to be active at once). In either case, each community must be censused every 6 months, so it may not take more than 6 months to complete all communities. The census must be completed (i.e., “locked”) at the household level before treatment can be given.

## Random Census Verification

A random re-census of households will be conducted at each study visit by supervisors, additional census team members, or local community monitors, as appropriate for the study site. Verification will be performed at the household level, with a minimum of 200 households being resurveyed during the 7 study visits (approximately 30 per visit). Each team must have at least one household census verified at each study visit.

Households will be selected using a different mechanism than the mechanism used by the original census. The primary method for selecting random households will be from aerial visualization (Google maps, AfriPop, etc.) If aerial visualization is not possible in an area, then another method for obtaining households can be used, such as a random walk.

Both census teams will use the same electronic template (i.e., no prior records at MORDOR 0, and the census records from the prior census at each follow-up visit). We will arbitrarily select a sample of communities stratified by census team. Once the census has been completed, the trial biostatistician will analyze the communities for verification.

The trial biostatistician will compare the results of the original census and the re-census to identify any discrepancies. The steering committee will determine any corrective actions once this comparison is made. At the very minimum, the site study coordinator will inform the original census team of the discrepancies and will conduct a refresher training session to minimize data collection errors.

- 1. **Verbal Autopsy**

Verbal autopsy questionnaires will be completed for all deceased children (aged 1-60 months) in the whole Nouna district.

**Staff**

Verbal autopsy interviews will be conducted by trained staff. Training will focus on conducting sensitive interviews with persons who may still be in mourning; discussion of verbal autopsy questions; reviewing the format of the paper and/or electronic questionnaires (including skip logic); and demonstration of the verbal autopsy technique on 5 mock deaths. The 5 first verbal autopsies will be observed by the study coordinator to ensure that proper procedures are followed. Each verbal autopsy interviewer will be responsible for a distinct geographic area, and will be responsible for regular contact with the key informant from each community.

**Identification of Deaths**

Deaths will be identified in 2 ways: from the biannual census, and from the key informant system. The CRSN data manager will provide a list of all deaths to the site study coordinator after each census. This list will include information on the deceased child’s name, age, gender, and unique identification number; community name; and father’s and mother’s names. The study coordinator will deliver this list to the appropriate verbal autopsy interviewer. The verbal autopsy interviewer will also keep a record of all deaths identified by the key informants. In each case, the key informant will report the community name, child’s name, and parents’ names, and the deceased child will be located in the census database.

**Questionnaire Administration**

The questionnaire will be administered as is done routinely for the HDSS. ^9^ The HDSS uses the WHO standard verbal autopsy questionnaire WHO-VA-2016 and cause of death will be assigned using InterVA 4.^10^ Questionnaires will be administered at the home of the deceased child. The informant will be the deceased child’s parent or guardian. If this person is not available, the verbal autopsy interviewer will try to arrange a time to return to interview this person. If the parent or guardian is not present on the third visit, they will complete the questionnaire by interviewing another family member, or as a last resort, a neighbor. All interviews will be completed in the local language. Our goal is to perform each verbal autopsy within 1 to 6 months of identification of death.  The child’s name and unique identification number will be recorded on the verbal autopsy record for identification purposes.

**Assigning the Cause of Death**

As recommended by the WHO, we will use automated methods to assign causes of death based on the verbal autopsy questionnaire, rather than physician review. We will treat all individuals under 4 weeks as one subpopulation, and individuals 1-60 months as a separate subpopulation for determination of cause-specific mortality fractions.

- 1. **Validating the mortality outcome**

No death registries exist at the health facility. All Deaths from our census will be investigated and will catch false positive deaths recorded during the census.

We will record all deaths happening at the health facilities to be able to catch deaths the field workers might have missed during the census.

The study coordinator will work closely with the health centers to be able to obtain a list of deaths occurring in his/her facility.

The Data Manager will be responsible for linking the deaths occurring at the health facilities with the census file, using the name, age, and village of deceased children.

Verbal autopsies will be performed on all deaths picked up by the census and also all deaths from 0-5 year-olds picked up by the health facility if not included on our census.

**Chapter 7: Registering Participants for Specimen Collection**

Samples will be collected with reference to age, gender, household, and community, but participant names will not be included in laboratory records to ensure privacy. Samples will thus not be associated with an individual’s name, but with a random identification number and/or QR code, masking laboratory personnel and preventing identification of individuals.

At each time point, each child selected for examination/specimen collection will be assigned an identification number for database anonymity.

For each community, the randomized registration list for examinations will be generated by the database and downloaded to the tablet using the mobile application.

After registration, the child and his/her guardian will be directed to the appropriate examination stations.

**Chapter 8: Blood samples**

We will collect:

**Protection of Examiner and Study Participant**

Prior to examinations at the blood station and the swab station, the examiner and tuber must be gloved. The examiner will put latex gloves on both of his/her hands prior to touching the participant and a new pair of gloves will be used for each participant in order to avoid transmitting infection between participants. Purell® Instant Hand Sanitizer will be available for hand sanitization when needed.

1. Thick and thin smears, assessed for malaria parasitemia and gametocytemia by CRSN microbiologists,
2. Microcuvettes, analyzed for hemoglobin in the field using a HemoCue analyzer (HemoCue AB, Ängelholm, Sweden), and
3. Dried blood spots, collected on FTA Elute cards (Whatman, Kent, UK; or appropriate substitution) and sent for laboratory testing for malaria using a nested PCR assay[^40^](#_ENREF_40) and/or TropBio cards (Tropbio Pty Ltd, QLD, Australia) for serologic testing.

The order of events at the blood collection station is: 1) finger prick; 2) blood spots on filter paper; 3) hemoglobin test; 4) thin and thick smears for malaria.

**It is important to handle all blood specimens with care**

**to minimize risk of infection**

**Wear gloves.** New gloves must be worn for each child.

**Clean spills.** In the event of a blood spill or splash, clean immediately with approved disinfectant (10% bleach or chlorhexidine solution) and wipe with absorbent material.

**Disposal of sharps.** All lancets must be disposed of properly in sharps containers.

**No food.** Food and drink are not allowed at the blood collection station.

- 1. **Fingerstick**

Inform the mother that her child’s finger will be pricked to obtain blood to test for malaria and anemia. Describe the finger prick procedure, reassure her, and answer all questions. The blood specimen should be collected as described below to minimize the discomfort of the child and to ensure sufficient blood volume collection.

A finger stick of capillary blood will be collected for thin and thick blood smears to assess for malaria, hemoglobin testing, and dried blood spots to be stored for later testing. Blood will be collected by a gloved health worker using aseptic technique. Gloves will be changed between each participant. The fingerprick or heelstick site will be disinfected using a 70% isopropyl alcohol swab.

**Fingerstick procedure:**

1. Prepare the disposable lancet. Use a NEW disposable lancet for each child. **Do not** re-use lancets!
2. The recorder will scan the child’s QR code, and place a random number sticker on the TropBio filter paper and the (right edge of the) slide.
3. Position the child for the finger stick. Make sure that the child’s right hand is warm and relaxed. Hold the child’s thumb, middle, or ring finger on his/her right hand (from the top of the knuckle to the tip of the finger) between your left thumb and finger and disinfect in small outward circles with an individually packaged alcohol wipe.
4. After the alcohol dries, use the thumb to lightly press the child’s thumb or finger from the top of the knuckle towards the fingertip to stimulate blood flow towards the sampling point (puncture site). For the best blood flow and least pain, prick the side of the thumb/fingertip, not the center. While applying light pressure towards the thumb/fingertip, hold the lancing device in your hand and prick the thumb/finger. If the finger prick is performed properly, a single prick should be sufficient to collect the required amount of blood.
5. Allow the blood to ooze out. Wipe away the first 2 or 3 drops of blood with gauze. If necessary, re-apply light to moderate pressure towards the thumb/fingertip (approximately 1 cm behind the site of the finger prick) until another drop of blood appears.

***Note:* Do not** squeeze forcefully. Avoid “milking” as it may dilute the blood with tissue plasma.

- 1. **Dried Blood Spots for Serology**

**Collecting the FTA Elute filter paper sample:**

1. Label the filter paper with a random number sticker.
2. Place 2-4 large drops of blood directly from the thumb or finger onto the large circle on the filter paper (if it is difficult to obtain 4 drops of blood, it is sufficient to collect 2 drops of blood).
3. Leave the filter paper to air dry for a few minutes, then place the sample into a small plastic bag along with a desiccant packet.
4. Leave the bag open for a few minutes more, and when the blood is **completely** dry, roll down the top of the bag and close with a piece of masking tape.
5. Store the filter paper samples (in small plastic bags) in a larger Ziploc bag. Keep all filter paper samples in a safe, dry place at room temperature.
6. Blood spots will be stored at room temperature in a locked cabinet in the study coordinators’ office.

**Collecting the TropBio filter paper sample:**

1. Label the filter paper with a random number sticker.
2. Grip the filter paper on the side without small circles. Place a droplet of blood directly from the thumb or finger onto five of the six circles, leaving the right one blank. Be sure to fill each circle completely.

*
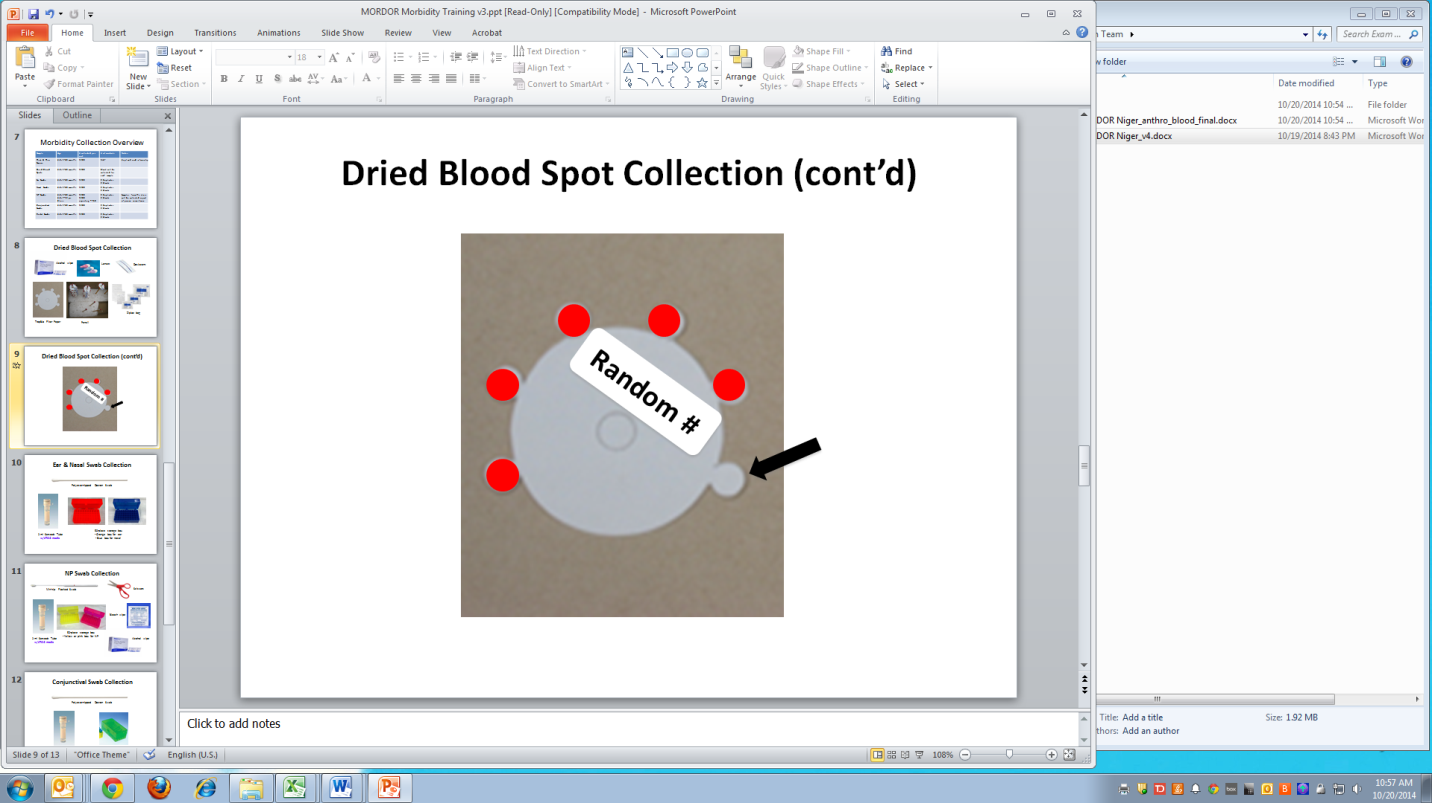
*

Area to hold the filter paper.
**Do not** touch the small circles.

Leave last circle blank

1. The recorder will scan the QR code.
2. Carefully slide the filter paper onto a pencil to air dry for at least an hour. There should be about 1 cm in between each sample. Secure the pencil into a Styrofoam surface in a box or container to protect from dust.
3. When the filter paper is dry, place each sample into a small zip plastic bag (individually). Place the small bags into a larger Ziploc bag with five desiccant packets.
4. Ensure the large Ziploc bag is sealed tightly, as moisture will damage the samples. Transport these filter paper samples to a freezer.

##
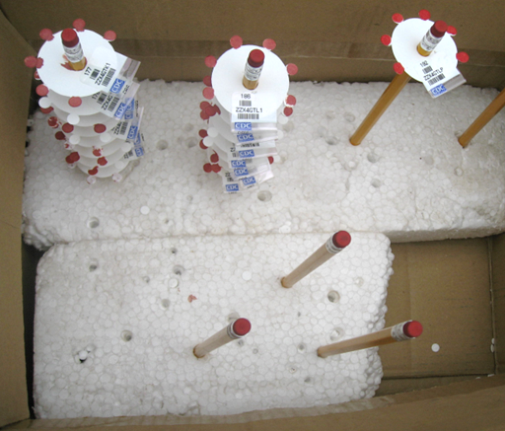
Set-up drying area for TropBio bloodspots

*Supplies:* pencils, Styrofoam, cardboard box, paper
- Place Styrofoam in cardboard box
- Put pencils inbox/container – space apart
- *Note:* When placing the blood spot samples on the pencil, space apart by ~2.5cm with pieces of paper in between each sample.

- 1. **Hemoglobin Test**

A portable spectrophotometer (HemoCue, Anglom, Sweden) will be used for hemoglobin testing.

**Set up HemoCue Analyzer**

1. Remove the HemoCue analyzer from the case. If a small battery symbol appears on the top right side of the display, the batteries are low. The HemoCue will still give accurate results, but it is strongly recommended to replace the batteries as soon as possible.
2. Pull the cuvette holder out to the loading position. Press and hold the left button until the display is activated (ALL symbols appear on display). The display will show the version number of the program, an hour-glass symbol and “Hb.” At this time, it will perform an automatic SELFTEST to verify the performance of the device. After 10 seconds, the display will show three flashing dashes and the HemoCue symbol. This means that the HemoCue has passed the SELFTEST and is ready for use. If the SELFTEST fails, an error code will be displayed.

**Collecting a blood sample for hemoglobin (HemoCue):**

1. Remove a cuvette from the container. Reseal the container immediately. (The recorder can help the examiner with this.)
2. When the blood drop is large enough, fill the microcuvette in one continuous process. **Do not refill!** If there is not enough blood to fill the microcuvette, you must start again with a new microcuvette. Wipe any excess blood from the sides of the microcuvette with clean gauze or a paper towel, but be careful to avoid touching the open end of the microcuvette so blood is not removed.
3. Look for any air bubbles in the filled microcuvette. If air bubbles are present, discard the microcuvette and obtain a new drop of blood using a new microcuvette. (Small bubbles around the edge of the microcuvette can be ignored.)
4. Place the filled microcuvette in the cuvette holder. Gently slide the cuvette holder to the measuring position to be analyzed immediately. (This **must** be performed within 10 minutes after filling the microcuvette.)
5. After 15 – 60 seconds, the hemoglobin value will be displayed. The examiner should read the hemoglobin value aloud so the recorder can enter it into the tablet computer. The value will remain on display as long as the cuvette holder is in the measuring position. The analyzer will turn off automatically after 5 minutes.

***Note:*** For children 6 months to 5 years, if the hemoglobin is <11.0 g/dL, the child is anemic. For children 5 – 11 years, if the hemoglobin is <11.5 g/dL, the child is anemic (WHO/UNICEF/UNU, 1997). If a child is found to be severely anemic, the examiner must refer him/her to the nearest health center for treatment.

1. Carefully dispose of the used microcuvette in the sharps container.
2. **At the end of the day:** Turn off the HemoCue analyzer. Press and hold the left button until the display reads OFF. The display should be blank.

**Cleaning the HemoCue Analyzer**

- 1. To clean, pull the cuvette holder to the loading position.
  2. Carefully press the small catch (upper right corner of the cuvette holder). Continue to press the catch and carefully rotate the cuvette holder as far left as possible, and then carefully pull the cuvette holder out of the analyzer.
  3. Clean the cuvette holder with alcohol or a mild detergent. Push a clean cotton tipped swab moistened with alcohol (without additive) into the opening of the cuvette holder and move from side to side 5 – 10 times. If the swab is dirty, repeat with a new clean swab until the cuvette holder is clean. A dirty cuvette holder may cause the HemoCue analyzer to display an error code.
  4. After 15 minutes (or less time, depending on the climate), you may replace the cuvette holder and use the analyzer. The cuvette holder must be completely dry before you replace it.
  5. Put the Hemocue analyzer back into its case.
  6. **Thick and Thin Smears for Malaria**

1. Label the slide with a random number sticker.
2. For the thick blood smear:
   1. Place a drop of blood in the center (1 cm from the edge of the slide) of a clean, dust-free, and grease-free slide.
   2. Spread the drop of blood evenly with a disposable wooden applicator or with another clean slide into a circle with a diameter of 1 cm.
   3. The blood smear should be about 1cm away from the edge of the slide. The correct thickness of a thick blood smear is one through which newsprint is barely visible when the blood is still wet.
3. For the thin blood smear:
   1. Place a smaller drop of blood on the slide.
   2. Using another slide angled at 45°, create a feathered edge before reaching the other end of the slide.
4. Allow the blood smears to air dry flat. Do not heat the slides, as this will damage the parasites. Be sure to protect the slide from dust and insects. Do not refrigerate slides, as this may cause the smears to detach from the slide during the staining procedure.
5. When dry, place the thick and thin blood smears into the slide box.
6. Smears will be transported at room temperature each day to a diagnostic facility near the study area.
7. Within 24 hours of thick and thin blood smear collection, the smears will be stained with 2% Giemsa stain for 30 minutes. (The thin smear will be fixed by submerging it in 100% methanol for 30 seconds and then let it air dry for 1-2 minutes prior to the Giemsa stain.)
8. Parasite density will be measured by a masked reader using a microscope at the diagnostic facility.

Smears will be stored at room temperature.

A rapid diagnostic test (RDT) could be substituted for thick smears if approved by the Steering Committee.

## Rapid Diagnostic Test

A rapid diagnostic test for malaria will be conducted at the final exams (CHAT 36).

Blood will be obtained from the finger prick site of the previous exams. A pipette will be filled with blood which will then be placed into the corresponding chamber on the test.

Three to 6 drops of the buffer solution will be placed into the chamber provided on the test.

The examiner will wait 15 minutes before reading the test.

Negative: A single red line appears under the letter “C” on the test. This is the control.

Positive: 2 red lines appear. One line under “C” on the test and a second line under “P.f” indicating *P. falciparum.*

If the result is positive, the child will be referred to the health center for treatment.

## Materials for Blood Collection

**Fingerprick**

Gloves

Disposable lancets

Alcohol wipes

Cotton balls

Gauze

10% household bleach or 4% chlorhexidine solution to clean spills

Absorbent material for spills

Sharps container

**Dried Blood Spots**

FTA Elute cards

Small zip plastic bags

Desiccant packs

Masking tape

Large Ziploc bags (handful)

TropBio circular cards

Small zip plastic bags

Desiccant packs

Large Ziploc bags (handful)

Materials for drying apparatus: 12 sharpened pencils, Styrofoam, empty cardboard box

**Hemoglobin Test**

HemoCue machine

Extra set of AA batteries

Cuvettes

Q-tips (handful)

**Thick and Thin Blood Smears**

Glass slides

Slide box

Rolls of random number stickers

**Rapid Diagnostic Test**

RDT

Solution

**Google Nexus 7**

External battery pack

# Chapter 9: Specimen Collection for Resistance Testing

## Population

We will collect nasopharyngeal and stool samples on a random set of 15 children aged 1-60 months from each of the Mortality Plus communities. Children will be selected from the current study census. The swabbing visits will occur after the census but **before** treatment. The randomized registration list of children will be provided to the site study coordinator before the swabbing visit. The Study Coordinator will give this list to the community for mobilization prior to examinations.

## 9.2 Nasopharyngeal Swabs

Nasopharyngeal swabs will be stored in DNA/RNA shield media by Zymo and STGG media, and standard microbiologic techniques will be used to isolate *S. pneumoniae* and test for resistance to azithromycin, penicillin, and clindamycin. Resistant isolates will be assessed for the most common genetic resistant determinants (*ermB* and *mefA*) using a PCR-based assay.[^41^](#_ENREF_41) Serotype will be assessed using a nested PCR reaction for the most common serotypes, followed by the Quellung reaction for any untyped isolates.[^42^](#_ENREF_42)

The examiner will:

1. Place a pediatric flocked swab with a nylon tip through the right nostril and down the nasopharynx of each participant. Note that if the swab is not perpendicular to the frontal plane of the face, it is likely not in the inferior turbinate.
2. Once you reach the nasopharynx, rotate the swab 180° as you remove the swab from the nose.
3. Place the swab in a tube containing 1.0 mL DNA/RNA shield media by Zymo or STGG (skim milk, tryptone, glucose, and glycerin) media, cut the handle off using sterile scissors, and close the cap of the tube with the swab immersed.
4. The nasopharyngeal swab samples in STGG will initially be stored in the field at 4°C using an insulated storage bag with Fisher brand ice gel packs, and then transferred to -20°C. The nasopharyngeal swab samples in DNA/RNA shield media will be stored in ambient temperature in the field. Then transferred to a refrigerator or freezer.
5. The scissors used to cut calcium alginate swabs will be sterilized with alcohol pads or cleaned with bleach wipes between participants. When collecting specimens in DNA/RNA shield, scissors will be cleaned between participants - first with bleach wipes, and then with alcohol pads.

Do not attempt to collect the NP swab if you are not successful after **three** attempts.

### **Materials for Swab Collection for Resistance Testing**

**Swabs**

NP specimens will be collected using sterile, individually-wrapped pediatric flocked swabs with a plastic swab shaft (manufactured by Copan).

**Sample Tubes**

All field samples for DNA testing will be collected into sterile 2.0ml microcentrifuge tubes, manufactured by Sarstedt®. (DNA-free tubes will be used for collection in DNA/RNA shield.)

**Cooler Bags with Frozen Ice Packs**

Insulated cooler bags will be used to carry samples to and from the field. In addition, frozen gel ice packs designed to thaw slowly will be used to maintain the temperature in the cooler bags during transport.

**-20°C Freezer**

A standard -20°C freezer located at the CRSN laboratory will be used strictly for the storage and freezing of ice packs and samples. This freezer is kept in a locked room on the grounds of the CRSN Laboratory, which is under 24-hour security guard supervision.

**-80°C Freezer**

A dedicated -80°C freezer located at the CRSN laboratory will be available for storage of study samples, including rectal and nasopharyngeal swabs.

### **Protocol for Tubing and Handling of Samples**

The tubing and handling protocol must be carefully followed in order to prevent contamination and ensure the safe transport of the samples back to the CRSN laboratory and/or to the US for processing. The person in charge of labeling, tubing, arranging, and handling the samples needs to perform this task in the most orderly and attentive manner.

1. Both hands of the tuber should be gloved at all times. The tuber's gloves only need to be changed when any potential contamination of the gloves occurs. The tuber opens the capped, hinged lid of a microcentrifuge tube, which has been labeled with the participant’s random identification number.
2. The swab is inserted by the examiner into the microcentrifuge tube held by the tuber. The swab shaft should only be inserted until the swab head is fully in the tube. The tuber will cut the swab shaft with sterile scissors.
3. The tuber should screw the cap of the microcentrifuge tube tightly, flick the tube to mix the sample with the media (for tubes with DNA/RNA shield media), and place it in the sample collection box, located in the cooler bag filled with frozen ice packs. The flap of the cooler bag should be closed between each patient. The cooler bag should be in as cool a place as possible in the field, in a shaded area out of the sun.

Upon returning from the field each day, the samples in STGG will be immediately taken to the CRSN laboratory and stored in a commercial -20°C freezer, reserved solely for storage of specimens and ice packs. All samples will be in sample boxes, labeled with the village name for easy future identification.

## Stool Samples

All study sites will collect either stool samples or rectal swabs for resistance testing, but methodology will depend upon the type of samples collected and consist of either culturing/microbiological testing or nucleic acid based testing.

**Rectal Swabs for Culturing/Microbiological Testing**

Rectal swabs will be collected and placed into Amies transport media or Norgen Stool Preservative. We will use standard microbiologic techniques to isolate *Escherichia coli*. Resistance to azithromycin, ampicillin, and co-trimoxazole will be determined. Isolates can be further classified into commensal and diarrheagenic subtypes using a multiplex PCR assay.^11^

**Stool Samples for Nucleic Acid Based Testing**

Stool specimens will be used to look for the presence of E. coli and macrolide resistant determinants typically associated with resistant strains of E. coli, using a resistome approach. This will be carried out by isolating DNA from stool specimens and detecting the presence of E. coli as well as genes associated with antibiotic resistance (i.e. *erm,* *mef, and mph* genes) via PCR assays. Other possible experiments may eventually include using PCR to detect the presence of toxins or virulence factors (i.e. *eae, stx, bfp-A,VT-1, VT-2*), which are linked to diarrheagenic E. coli strains, and exploring the complete microbiome of the stool specimens using DNA and RNA sequencing.

### **Stool Specimen Collection**

**Rectal Swab Collection for Culturing/Microbiological Testing**

The test will require that the child’s parent and examiners work together to obtain a good sample. Is it important to describe the test to the parent so that they can best assist with keeping the child still during the procedure, if necessary.

In place of stool specimens, rectal swabs can be collected in the following way:

1. Put on a clean pair of gloves.
2. Partially open the fecal swab package and remove the top section of the collection vial (this can be discarded).
3. Position the child:
   - Lie the child on his/her back, hold legs in the air (it is useful to have assistance).
   - Or have the child lay on his/her stomach across the mother/guardian’s lap
4. Remove the swab from the package. Take care that the cotton tip is not touched. If it is touched, throw the swab away and begin with a new one.
5. Insert the tip of the swab into the child’s anus only as far as needed to contact fecal material (1-3cm) and rotate 180 degrees. The tip should be a brownish color when removed.
6. Place swab into the preservative in the collection tube. Make sure the swab is fully submerged in the liquid preservative and then break the swab off using the pre-scored breaking point.
7. Screw the cap back on the tube and make sure that it’s tightened. Wrap the area where the cap meets the tube with Parafilm to ensure that the sample will not leak, and then place the tube into the appropriate sample box.
   - If the swab cannot be broken off while the tip is fully submerged in the liquid, try twirling the swab in the liquid first (to release the contents of the sample into the preservative) before breaking it off. Avoid rubbing the sample on the tip of the swab off on the side of the tube where there is no liquid.
8. Place a random number label on the collection tube.
9. Place the tube the rectal swab container.
10. **Swab storage for Genetic analysis:** Store samples at room temperature. According to the manufacturer, the preservative in the tube will preserve DNA for 5 months at room temperature (7 days for RNA), and thereafter can be frozen (-20°C or -80°C) for long-term storage.

**Stool Specimen Collection for Nucleic Acid Based Testing**

For the study participant/parent of the child:

1. Collect the initial stool specimen on a piece of plastic.
2. Transfer a few heaping spoonfuls of the fresh stool into the smaller, 4 oz disposable plastic container that has a locking lid, using the spoon provided. Return this to the trained field worker.

For the trained field worker:

1. Wearing fresh gloves, carefully place a portion of the stool sample from the disposable 4 oz plastic container into a labeled Norgen Stool Nucleic Acid Collection and Transport Tube (15 ml collection tube that contains preservative), using the small spatula that is attached to the tube’s cap. Fill up to the line as indicated by the tube.[^44^](#_ENREF_44) Make sure to spoon the stool into the tube without touching the rim or outside of the tube to avoid any contamination.
2. Once the stool sample has been added, place the cap tightly back onto the tube.
3. Mix gently until the stool is well submerged under the preservative. Do not shake the tube up and down, just gently swirl.
4. Wrap the lid of the tube with a piece of Parafilm to seal it.
5. Place the tube into the storage box and store at room temperature.
6. Once the final stool sample has been collected in the Norgen Stool Nucleic Acid Collection and Transport Tube, wrap up the initial stool sample in the large receptacle container using the plastic lining and properly dispose of it. Also, dispose of the stool sample in the 4 oz plastic container and the spoon that was used.

### **Materials for Stool Specimen Collection**

**Rectal Swab Collection for Culturing/Microbiological Testing**

**Swab**

An individually-wrapped Copan flocked swab with a plastic shaft will be used to collect the rectal swab and then placed into a Stool Nucleic Acid Collection and Transport Tube containing Norgen Stool Preservative or Amies Transport Medium.

**Sample Tube with Media**

The specimen will be in a sterile Stool Nucleic Acid Collection and Transport Tube containing Norgen Stool Preservative or Amies Transport Medium with a cap that will be tightened firmly.

**Stool Specimen Collection for Nucleic Acid Based Testing**

**Plastic Lining**

Each participant will be given a piece of plastic that will be placed on the ground to collect the initial stool sample.

**Spoon**

Wooden medical spoon used to transfer a portion of the initial stool specimen to the small plastic container, which will be brought to the trained field worker by the parent.

**Small Plastic Container**

4 oz disposable plastic container with locking lid used to transport a portion of the initial stool specimen to the trained field worker.

### **9.3.3 Protocol for Fresh Frozen Stool Collection**

**Materials**

- Stool specimen (10-20 grams or ml)
- Pre-printed PID labels (4 plus 1 extra)
- Plastic disposable transfer pipette for liquid stools
- Cotton-tipped wooden stick
- Wide-mouthed plastic container suitable for collecting stools
- Wooden spatula
- Frozen ice packs
- Cold box
- Tube rack
- Disposable latex gloves
- Disposable diaper
- Sealable plastic bags
- Plastic spoon
- Pen
- Stool Field Collection Form (SFC)

**Collection Procedure**

The stool sample is to be collected within a two day window of the scheduled time. Even with the best of efforts the field worker fails to collect stool sample within 2 days window, field worker may visit the home up to 5 days beyond that +2 time frame.

For collection of Stool, inform child’s/participant’s primary caretaker/ participant one day before planned stool collection and request caretaker to collect the first available fresh stool sample from the child on the morning of the planned visit.

The mother / participant’s primary caretaker/ participant should be provided with the labeled stool container, diaper (for infants), cold box, ice packs, gloves, plastic spoon, and 2 plastic bags the evening before planned stool collection. There should be enough ice packs in the cold box to keep it cold for up to 8 hours.

Instruct the caretaker/ participant to use the plastic spoon to collect 3-4 spoons of stool within 20 minutes of defecation and place it in the stool container, close the lid tightly, and place the container in the plastic bag.

**Temporary Storage and Transport Procedures**

Instruct the caretaker to place the plastic bag with the stool in the cold box immediately after collection (maximum time: 20 minutes). Collect the stool specimen as soon as possible and document if the sample was in a cold environment on the requisition CRF. Also document if specimen is acceptable (estimated quantity, lid closed, and no leakage)

**Processing**

Label the original stool container with SID labels. Write the date of collection (DD/MM/YY) and time of collection (hh:mm; 24 hour time scale) on the label.

**Collection and Transport Procedures for Microbiome analysis**

Initial collection of fecal samples should be made in a suitable sterile container after which smaller aliquots of fecal material should be transferred by the field worker (*within 20 minutes of defecation)* into pre-labeled, sterile 2ml cryo-safe tubes*.* Tube labels should minimally include the Participant’s Sample ID (SID) and the date of collection, or as specified by the BEED manual of procedures.

Wearing clean, disposable latex gloves, fill each 2 ml cryo-vial approximately one-half to two-thirds full using a sterile spatula and cap tightly. Do not add any buffers, preservatives or additives to the sample. [Note that use of screw cap vials and not overfilling them minimizes the potential for cross-contamination of samples during transport and storage].

Immediately place capped vials into liquid nitrogen pre-charged ‘dry shippers’ (for transport back to the laboratory. Specimens should be transferred to dry shippers within 20 minutes of defecation. [Note: dry shippers can be reused between charges so long as they are checked each morning following the manufacturer’s instructions to ensure sufficient liquid nitrogen is present to complete the intended sampling needs for the day].

Upon return to the laboratory, empty the vials from the dry shipper into a bucket of dry ice to prevent thawing while sorting and transferring the vials to 9x9 freezer boxes for longer term storage and transport.

## Quality Control Measures for Specimen Collection

**Negative Field Controls**

Negative field control swabs for NP and stool will be taken in each community to assess for contamination: one control swab each (NP and stool/rectal) are taken before specimen collection begins in a community; and another (NP and stool/rectal) upon completion of specimen collection.

- - - 1. For each negative field control, the examiner will open a new swab as described above.
      2. Wave the swab in the air, without making contact with anyone/anything.
      3. Tube the swab in media, as described above.

**Duplicate swabs**

Duplicate NP swabs and rectal swabs/stool specimen will be collected from two children per community.

# Chapter 10: Training

## **Standardization**

The research team will work together prior to the baseline visit to standardize all study procedures. We will review the format, general logistics, and procedures for the house-to-house census. The importance of capturing the vital status of every individual in the study area, including individuals not on the previous census (i.e., new births, deaths and migrations) will be stressed. The importance of capturing those individuals who were born and died in the time period between two censuses will also be highlighted. Census workers who have successfully completed the training will be certified, although certification can be revoked on subsequent quality control checks. Ongoing training activities (before each biannual census) should consist of didactic classroom instruction and mock census activities, followed by in-field training, reviewing the use of the electronic data capture, including charging devices and troubleshooting technical problems.

**Chapter 11:** **Sample Organization, Transport, and Storage**

## **De-identification**

All specimens will be labeled in the field with a random identification number linked to the census in the electronic data capture system, but to facilitate masking, only the CRSN DCC will have access to the key linking the ID with census information. Age, gender, and community of residence will be available for each specimen, but names will be kept confidential. Therefore, all specimens will be de-identified.

- 1. **Specimen Transport**

After sample collection, samples from the field will be transported to the CRSN laboratory for storage and processing.

During any international specimen transport, the temperature of the shipper boxes will be documented by a temperature recording device.

**Blood Samples**

Blood smears (thin and thick) will be transported at room temperature to the CRSN laboratory. FTA Elute cards will be transported at room temperature and stored at the health clinic before being transported for processing. TropBio cards will be transferred on ice to the health center and stored at -20°C prior to shipment.

**Swabs**

Swabs in STGG media will be initially stored in the field at -4°C using a closed, insulated container until arrival at a securely locked freezer at -20°C. Swabs in DNA/RNA Shield media will be stored in ambient temperature in the field and then transferred to a refrigerator or freezer.

**Stool Samples**

**Rectal Swabs for Culturing/Microbiological Testing**

Rectal swabs preserved in Amies transport medium should be refrigerated until processed. If specimens will be kept more than 2 to 3 days before being cultured, it is preferable to freeze them immediately at -80°C. It may be possible to recover pathogens from refrigerated specimens up to 7 days after collection; however, the yield decreases after the first 1 or 2 days. Frozen specimens should be transported on dry ice.

**Stool Samples for Nucleic Acid Based Testing**

Specimens preserved in Norgen Stool Nucleic Acid Collection and Transport Tubes can be left at room temperature for 7 days (if preserving RNA) or up to 5 months (if preserving DNA). Specimens can also be transported in the preservative at room temperature. If the samples will be kept for long term storage, they can be placed in a -20°C or -80°C freezer.

- 1. **Specimen Storage**

Sample storage will occur in two stages: short-term and long-term.

- - 1. **Short-term Sample Storage**

Samples will be labeled with study ID only and are unidentifiable without access to the study database. All samples will be transported to the CRSN laboratory for storage and processing. A subset of samples will be shipped to UCSF to process core samples and any secondary processing.

- - 1. **Long-term Sample Storage**

All samples processed by the CRSN laboratory will be stored in the -80°C freezer for at least 5 years. A subset of de-identified samples from Burkina Faso will be shipped to UCSF for longer-term storage at the UCSF Oyster Point Facility, which is designed particularly for secure long-term (5 years) storage of biological specimens, at -80°C for future analyses by CRSN and UCSF investigators and other interested parties.

- 1. **Catalog Specimens**

We will create a list of study data and specimens, including the age, gender, village identification number, treatment assignment, whether treatment was received, vaccination record, and symptom questionnaire. We will also list the date of collection and transport, and the storage conditions while in the field and while banked at UCSF. This will facilitate identification of specimens for future analyses.

# Chapter 12: Study Medication

Children aged 1-59 months on the current census will be offered weight- or height-based, directly observed, oral suspension (azithromycin or placebo) every 6 months for 3 years (as performed in trachoma programs) at each study site. At the final phase of CHAT, all children 1-59 months will be offered azithromycin. Children under the age of 12 months or not able to stand will be weighted. In addition to being at least 1 month of age, children should weigh at least 3.8 kg to be eligible for treatment. This ensures that mistakenly aged or premature infants won't be treated. These infants will be eligible for treatment at the subsequent distribution, approximately 6 months later. The mortality application will not provide a dose for children weighing <3.8 kg.

We will monitor adverse events following mass treatments as described in the adverse events section. The treatment and monitoring schedule for all study arms is shown in Table 1.

## 12.1 Study Medication Description (from Pfizer, Inc.)

**Azithromycin**

Zithromax® for oral suspension is supplied in bottles containing azithromycin dehydrate powder equivalent to 1200mg per bottle and the following inactive ingredients: sucrose; tribasic anhydrous sodium phosphate; hydroxypropyl cellulose; xanthan gum; FD&C Red #40; and flavoring including spray dried artificial cherry, crème de vanilla, and banana. After constitution, a 5mL suspension contains 200mg of azithromycin.

## Dosage Information

Azithromycin and placebo will be administered as a single dose, in oral suspension form for children. Dosing will follow the WHO recommendations for treatment of active trachoma:

- Single dose of 20mg/kg in children (up to the maximum adult dose of 1g)
- Height-based dosing of children ( this dosing method is supported by the WHO)

Individuals who are allergic to macrolides/azalides will not be treated.

## Medication Procurement/Donation

Azithromycin (Zithromax®) and the placebo have been donated by the Pfizer Corporation. There will be no costs to acquiring the study medication. Pfizer, Inc. will ship azithromycin and placebo directly to the study sites. Representatives of the study site will manage the customs process and transport the medication from the port to storage sites.

## Medication Quality Control

Study medication will be shipped directly from Pfizer and stored at CRSN prior to use. The study coordinator and other staff will regularly check and record the study medication expiration dates. We will strictly monitor expiration dates on the medication containers and all expired study medicine will be discarded appropriately.

## Antibiotic Distribution & Monitoring Coverage

After the MORDOR 0 census and monitoring/collection is complete, treatment (azithromycin and placebo) will be administered to all eligible community members per study protocol. Teams will participate in training exercises regarding drug/placebo distribution and recording techniques prior to each treatment cycle. Training will be in accordance with the Zithromax Program Manager’s Guide from the International Trachoma Initiative.

During mass drug administration, distribution team members will use tablet computers equipped with an electronic data capture system to seek out each eligible child on the census, administer antibiotic or placebo, and record whether or not each person has been treated. The distribution team will document individual reasons for not being treated (e.g. death, temporary absence, permanent migration, refusal of treatment, etc.). Consumption of medication will be directly observed and the dose distributed will be documented in the electronic data capture system.

We will estimate antibiotic coverage from the most recent biannual census records, aiming for treatment of 80% of children. At the end of each treatment round, the DCC will identify any children who have missed 2 or more consecutive treatments, and relay this information to the study coordinator. Census teams will discern the reason for missing treatments (including unrecorded death) at the next scheduled census. This system will serve as a quality control mechanism to reduce the number of false negative deaths in the study.

## Adverse Reactions/Side Effects

Azithromycin is generally well-tolerated. The most common side effects of azithromycin are diarrhea, nausea, abdominal pain, and vomiting, each of which may occur in fewer than one in twenty persons who receive azithromycin. Rarer side effects include abnormal liver function tests, allergic reactions, and nervousness. Diarrhea due to *Clostridium difficile* has been reported in rare cases.

During the consent process, the common adverse reactions that may occur will be explained to parents/guardians and they will be advised to communicate adverse events to CRSN study staff immediately. If, for any reason, the participant needs further care, they will be referred to the nearest health center for examination and treatment.

The trial sites will be masked to outcomes, so the responsibility for monitoring interim analysis will fall on the DSMC. Statistical monitoring is discussed in the Statistical Analysis Plan. The Data Safety and Monitoring Committee (DSMC) will be given authority to discontinue treatments at any time if there is evidence of unexpected harm.

## Adverse Events Systems

Both active and passive monitoring systems for adverse events are in place for this study, and these monitoring activities will specifically include (but will not be limited to) treated 1-6 month olds. We will monitor adverse events following mass treatments actively at each follow-up census and during a house-to-house survey of all 1-6 month olds in a random selection of azithromycin and control communities.

### **Passive Adverse Events Monitoring**

We will implement a passive monitoring system during the treatment phase, by instructing parents to report any adverse events in the two weeks following each mass azithromycin distribution to a local healthcare provider. Children will be referred for follow up care on a case-by-case basis.

### **Active Adverse Events Monitoring**

**Infant Adverse Events Survey**

To identify any adverse events associated with mass treatment, the research team will randomly select 48 study communities (12 per arm) to participate in an adverse events survey. This survey will be performed by the census workers masked to treatment arm, approximately 2 weeks after a mass medication distribution during the first phase only (CHAT 0). During the survey, adverse events will be elicited only for study participants aged 1-6 months at the previous census. A structured questionnaire will be performed to elicit dangerous side effects, followed by an open-ended question. Specifically, we will ask the primary caregiver about the following symptoms during the time since the previous antibiotic distribution: abdominal pain, vomiting, diarrhea, constipation, hemorrhoids or rash. We will only collect this infant adverse events survey for the first phase. The rest of the phases of the trial will not collect this information.

**Training**

The household survey team will be the same individuals who conducted the census. They will be trained in survey administration methods, including:

1. Obtaining informed consent
2. Accurately selecting the appropriate households to interview
3. Remaining neutral when asking questions (i.e. asking the question exactly as it is written on the paper in a neutral tone of voice, so as not to lead the respondent or introduce bias)

**Serious Adverse Events**

Any serious adverse events (SAE) will be reported to Pfizer. An **IIR SAE Form** (*Investigator-Initiated Research Serious Adverse Events Form*) will be completed for each event. (See Appendix for form and complete instructions.)

According to Pfizer, an SAE is any adverse event that:

- Results in death
- Is life-threatening (i.e., causes an immediate risk of death)
- Requires inpatient hospitalization or prolongation of existing hospitalization
- Results in persistent or significant disability or incapacity
- Results in a congenital anomaly or birth defect

Or that is considered to be:

- An important medical event

All community residents will be advised to alert a village health worker if they experience, within one week of mass treatment, a serious adverse event (by the preceding definition). An SAE report must be submitted for all deaths in the study – regardless of the time of treatment. The local health worker will report to the study coordinator; who must, within 24 hours, submit a Pfizer **IIR SAE Form** to [mordor.burkina.sae@gmail.com](mailto:mordor.burkina.sae@gmail.com). AS and TL will review, and forward to Pfizer and/or the Medical Monitor, as appropriate. SAEs must be submitted to Pfizer within 24 hours of receipt from the on-site coordinator. AS and TL will also forward SAE to DSMC if meets criteria of being possibly related to study drug. The reporting of any serious adverse event will follow national procedures in Burkina Faso.

- In the event of serious events, the CSPS will contact the study doctors on the same day. The patient will then be evacuated to an appropriate level of care for management.
- The declaration will be made to the National Agency for Pharmaceutical Regulation in accordance with the regulations and within the deadlines (7 days in the event of death or life-threatening prognosis, 15 days in other serious and unexpected cases, 15 days in new facts) at [pharmacovigilance.burkina@sante.gov.bf](mailto:pharmacovigilance.burkina@sante.gov.bf)
- The ethics committees will also be informed of the occurrence of this event within the same time frame.

Deaths that are reported to the study team outside of the biannual census (primary outcome) will be reported as an SAE to Pfizer. Note that deaths identified via the biannual census, which constitute the primary outcome, will not be reported as SAEs. Deaths that are reported to the study team as part of the biannual census will be reported to Pfizer in aggregate, not by arm, on a quarterly basis.

### **Adverse Events Data**

We will keep records and report all adverse events of azithromycin to the DSMC. We will report both efficacy and side effects of azithromycin separately for the 1- 6 month old age group. For any “sudden deaths” believed to be associated with azithromycin treatment, key informants will immediately notify the verbal autopsy interviewer via SMS message or another appropriate form of rapid communication. Reporting of non-serious adverse events will follow national procedures in Burkina Faso, as well. In the event of a non-serious adverse event, the CSPS will process the cases and the national reporting form will be completed. Any adverse event occurring during the trial will be covered by the study free of charge.

## 12.8 Supply issues

If a study site runs out of a treatment letter, a request should be sent to [mordor.burkina.tx@gmail.com](mailto:mordor.burkina.tx@gmail.com) to request a replacement for the community in question. This is not blanket permission to substitute one letter for another – if there are several communities for which the assigned treatment has run out, a separate request must be made for each community.

The study site coordinator will make the request; TCP will determine the replacement letter, a member of the DCC will make the change(s) in the database.

The field team must log out of the MORDOR mobile app and log in again for the changes to take effect on the front end. The replacement treatment letter will then appear in the app.

# Chapter 13: Protection of Human Subjects

Before the study begins, the research team will obtain formal ethical approval from their respective ethics committees as well as national ethical approval in Burkina Faso. In addition, staff will approach community leaders to describe the study and answer any questions. Study staff will proceed only if local leadership consents to participate. Verbal consent will be collected from the village leadership. We will also obtain verbal consent from the head of household to be able to perform the census. To be able to examine and treat the children living in the household, we will obtain written informed consent from a parent or guardian. This written consent will contain information regarding all study activities with patient contact: examinations, and treatments. We will collect one written consent form per child the first time we enroll the child. The subsequent visits we will explain the study to the parent/guardian of the child but we will only obtain verbal consent. Children will be included in the study only following the receipt of the written consent from a parent or guardian. If, at any time, a parent or guardian elects to withdraw a family member from the study, they will be free to do so. Individuals who withdraw will be offered the same medical treatment outside the study.

Children with wasting, stunting, malaria, or anemia will be referred for appropriate treatment by trained study personnel, at the nearest health center.

## Institutional Review Board Approval

**UCSF Committee on Human Research**

UCSF’s Committee on Human Research will annually review study protocol for ethical approval.

**CRSN Comité Institutionnel d’Ethique**

The study protocol will be reviewed and granted ethical approval by the Comité Institutionnel d’Ethique at the CRSN headquareters before any patient-related research activities begin.

**National Health Ethics Committee of Burkina Faso.**

The study protocol will be reviewed and granted ethical approval by the National Health Ethics Committee of Burkina Faso before any patient-related research activities begin and annually.

## Informed Consent

First, the chairman of each village will be asked for permission to include the village in the study. Additionally, the study will be discussed with all adults in the village by team members who speak the local language(s).

Informed consent scripts will be translated into local languages before the study can begin. Consent scripts will then be back-translated by a different party to ensure comprehension. Consent scripts will be submitted and approved by national IRB committees in Burkina Faso prior to study implementation. Then they will be read aloud to each study participant (and his/her parent/guardian) by a team member who is a native speaker of the local language to ensure that they understand the risks and benefits of participating in all study activities. Young adults and children under 18 years of age, who cannot give consent by law, will be included in the study only following the receipt of written informed consent from a parent or guardian. If, at any time, a parent or guardian elects to withdraw themselves or a family member from the study, it will be made clear that they will still be eligible for treatment.

## Risks and Benefits of Study Procedures

### **Verbal Autopsy**

As verbal autopsy requires a family member to answer questions about a deceased loved one, he or she might experience emotional stress and grief related to the death of the child. Interviewers will be trained to address these situations appropriately with awareness of the cultural context before they are allowed to conduct these verbal autopsies. If the family member is in need of a mental health intervention, referrals will be made by the interviewer.

### **Swabbing Procedures**

There are minimal risks to the participant who receives nasopharyngeal, and nares swabbing. Participants may experience some temporary discomfort, but the swabbing involves minimal risk. Any adverse effects, such as nose-bleeds, will be treated immediately by the examiners. Other health care will be provided at no cost to the study participant if necessary to address a study-related adverse health event.

### **Stool Collection**

Stool samples have been collected in this setting before, with essentially no risk to participants.

### **Blood Testing**

Blood testing will include a pin prick to the finger or heel.  The major risk of this procedure is infection at the puncture site, though using aseptic technique will minimize this occurrence. Individuals in these communities are familiar with this procedure because all children who present at a health center with fever are offered the pinprick for a malaria thick smear.

### **Anthropometric Measurements**

There are minimal risks associated with the measuring board, scale, or MUAC tapes aside from anxiety during the measurements. Examiners will do their best to ensure that the parent/guardian of the child understands the process of assessing anthropometric measurements.  The examiners will attempt to minimize discomfort for all study participants before, during, and after the measurements are taken. Children with wasting, stunting, malaria, or anemia will be referred for appropriate treatment at the nearest health center.

# Chapter 14: Study Monitoring

The project will be continuously monitored by the supervisory team, which will consist of members from CRSN and UCSF. The supervisory team will conduct regular monitoring visits to study site locations, with UCSF team members accompanying CRSN team members at least biannually.

# Chapter 15: Data and Safety Monitoring Committee Charter

This Charter is for the Data Safety and Monitoring Committee (DSMC) for *Mortality Reduction after Oral Azithromycin II Burkina Faso (*MORDOR II Burkina): OPP1187628.

The Charter will define the primary responsibilities of the DSMC, its relationship with other trial components, its membership, and the purpose and timing of its meetings. The Charter will also provide the procedures for ensuring confidentiality and communication, statistical monitoring guidelines to be implemented by the DSMC, and an outline of the content of the Open and Closed Reports that will be provided to the DSMC.

## Primary Responsibilities of the DSMC

The DSMC will be responsible for safeguarding the interests of trial participants, assessing the safety and efficacy of the interventions during the trial, and monitoring the overall conduct of the trial. The DSMC will provide recommendations about stopping or continuing the trial. To contribute to the integrity of the trial, the DSMC may also formulate recommendations relating to the selection/recruitment/retention of participants, to protocol-specified regimens, and the procedures for data management and quality control.

The DSMC will be advisory to the trial leadership group, hereafter referred to as the Steering Committee (SC). The SC will be responsible for promptly reviewing the DSMC recommendations and determining, whether to continue or terminate the trial, and to determine whether amendments to the protocol are required. If needed, the DSMC may seek the advice of a content expert outside of the committee.

- 1. **DSMC Membership**

The DSMC is an independent multidisciplinary group consisting of epidemiologists, biostatisticians, bioethicists, and clinicians that collectively has experience in the management of infectious diseases and in the conduct and monitoring of randomized clinical trials including subsaharan Africa.

- 1. **Conflicts of Interest**

The DSMC membership has been restricted to individuals free of apparent conflicts of interest. The source of these conflicts may be financial, scientific, or regulatory. Thus, neither study investigators nor individuals employed by the sponsor, nor individuals who might have regulatory responsibilities for the trial products, are members of the DSMC.

The DSMC members will disclose to fellow members any consulting agreements or financial interests they have with the sponsor of the trial, with the contract research organizations (CRO) , or with other sponsors having products that are being evaluated or that are competitive with those in the trial. The DSMC will be responsible for deciding whether these consulting agreements or financial interests materially impact their objectivity.

The DSMC members will be responsible for advising fellow members of any changes in any of the membership requirements that occur during the course of the trial. It may be appropriate for DSMC members who develop significant conflicts of interest resign from the DSMC.

DSMC membership is to be for the full duration of the trial. If any members leave the DSMC, the SC, in consultation with the DSMC, will promptly appoint a replacement.

- 1. **Timing and Purpose of the DSMC Meetings**

**Organizational Meeting**

The initial meeting of the DSMC will be an Organizational Meeting. This is during the final stages of protocol development and the purpose is to provide advisory review of scientific and ethical issues relating to study design to discuss the standard operating procedures and to discuss the format and content of the Open and Closed Reports that will be used to present trial results.

The Organizational Meeting will be attended by all DSMC members, lead trial investigators, and the trial biostatistician. The DSMC will be given the drafts of the trial protocol, the Statistical Analysis Plan, the DSMC Charter, and the current version of the case report forms. At subsequent meetings, committee members will receive Open and Closed Data Reports.

**Formal Interim Analysis Meetings**

One or more ‘Formal Interim Analysis’ meetings will be held to review data relating to treatment safety and efficacy, and quality of trial conduct. There will be at least two interim decisions to be made by the DSMC, at approximately 12 months and 24 months into the study.

## Procedures to Ensure Confidentiality and Proper Communication

To enhance the integrity and credibility of the trial, procedures will be implemented to ensure the DSMC has access to all emerging information from the trial regarding comparative results of efficacy and safety, aggregated by treatment arm.

**Closed Sessions**

Sessions involving only DSMC members and, where appropriate, those unmasked trial investigators (on the Data Coordinating Committee) who generate the Closed Reports (called Closed Sessions) will be held to allow discussion of confidential data from the trial, including information about the relative efficacy and safety of interventions.

At a final Closed Session, the DSMC will develop a consensus on its list of recommendations, including that relating to whether the trial should continue.

**Open Session**

In order for the DSMC to have access to information provided, by study investigators, or members of regulatory authorities, a joint session between these individuals and DSMC members will be held between the Closed Sessions.

**Open and Closed Reports**

For each DSMC meeting, Open and Closed Reports will be provided. Open Reports, will include data on recruitment and baseline characteristics, pooled data on eligibility violations, and completeness of follow-up and compliance. The study statistician (TCP) will prepare these Open Reports.

Closed reports, available only to those attending the Closed Sessions of the meeting, will include analyses of primary and secondary efficacy endpoints, including subgroup and adjusted analyses, AEs and symptom severity, , and Open Report analyses that are displayed by intervention group. These Closed Reports will be prepared by the study biostatistician.

The Open and Closed Reports should provide information that is accurate, with follow-up that is complete to within two months of the date of the DSMC meeting. The Reports should be provided to DSMC members approximately three days prior to the date of the meeting.

**Minutes of the DSMC Meeting**

The research team will prepare minutes for the open portion of the meeting, including the DSMC’s recommendations.

**Recommendations to the Steering Committee (SC)**

At each meeting of the DSMC during the trial, the committee will make a recommendation to the Steering Committee to continue or terminate. This recommendation will be based primarily on safety and efficacy considerations and will be guided by statistical monitoring guidelines defined in this Charter.

Recommendations to amend the protocol or conduct of the study made by the DSMC will be considered and accepted or rejected by the SC. The SC will be responsible for deciding whether to continue or to stop the trial based on the DSMC recommendations.

The DSMC will be notified of all changes to the protocol or to study conduct. The DSMC concurrence will be sought on all substantive recommendations or changes to the protocol or study conduct prior to implementation.

The SC may communicate information in the Open Report to the sponsor and may inform them of the DSMC recommended alterations to study conduct or early trial termination in instances in which the SC has reached a final decision agreeing with the recommendation. The SC will maintain confidentiality of all information it receives other than that contained in the Open Reports until after the trial is completed or until a decision for early termination has been made.

## Statistical Monitoring Guidelines

The SC will propose statistical rules for a futility stopping rule (requested by the sponsor) and an efficacy stopping rule at the first DSMC meeting. A decision will be made whether the efficacy stopping rule is appropriate for the relatively short, 2-year study.

## DSMC Contact Information

**Table 5**: DSMC Contact Information

| Allen Hightower, Chair | [awh1953@gmail.com](mailto:awh1953@gmail.com) |
| --- | --- |
| Amza Abdou | [dr.amzaabdou@gmail.com](mailto:dr.amzaabdou@gmail.com) |
| Jackie Glover | [Jackie.Glover@ucdenver.edu](mailto:Jackie.Glover@ucdenver.edu) |
| Wafaie Fawzi | [mina@hsph.harvard.edu](mailto:mina@hsph.harvard.edu) |
| Miriam Laufer | [mlaufer@som.umaryland.edu](mailto:mlaufer@som.umaryland.edu) |

# Chapter 16: Data Collection, Management, and Security

## Scope of Data

Mortality and morbidity data will be collected in this trial. Mortality data includes: census, mortality, and treatment. Morbidity and resistance data includes the following: census, mortality, treatment, and morbidity assessments.

**Mortality Data**

Trained census workers will collect census data on all households in the study sites (name, birthdate, age, gender of all household members) and keep track of births, deaths, and migration of children eligible for treatment. In addition to biannual census updates, trained community health workers and study supervisors will conduct WHO verbal autopsy interviews through the duration of the study to provide information on the cause of death. Trained distribution teams will collect data on treatment status and dose given to all study participants, if treatment is provided apart from the time of census.

**Mortality-Plus Data**

Trained health workers will collect data on core morbidity assessments such as blood samples (thick smears and dried blood spots for malaria, microcuvettes for hemoglobin), stool samples, and nasopharyngeal swabs. Note that for de-identification purposes a random number sticker will be affixed to each specimen collected. In addition, before sample collection, parents or guardians will be asked a standardized series of questions to determine whether the child has had recent fever, cough, or diarrhea. Clinic-based case finding will be conducted at local health clinics, which will involve transcription of health records.

Certain morbidity assessments will be entered into handheld mobile devices at the time of the examination (e.g. hemoglobin, responses to symptom questionnaire), while lab results for thick smears, dried blood spots, nasal, nasopharyngeal, and stool specimens will be entered after confirmation.

- 1. **Data Storage, Management, and Security**

Data will be recorded electronically using handheld mobile devices with custom-made software applications and uploaded daily onto a secure, password protected, central server. Rapid transfer of electronically captured data will allow nearly real-time monitoring of activity at the study site. All handheld devices and data entry coordinating centers will be password protected, and all changes in data will be noted, including the date of the change, and the person who made the change. To ensure the quality of the data, we will conduct training sessions before each biannual census where needed. The central database application will use hard disk encryption and physical protection of the server (which is to be maintained in a locked room accessible only to authorized personnel). The database will be based on mySQL (which supports standard SQL queries). Data will be backed up off site (providing integrity in case of the physical loss of the server). Data will never be deleted from mobile capture devices until at least one offsite backup has been completed. Data security during electronic transfer will be achieved through use of the Advanced Encryption Standard (AES).

- 1. **Data Monitoring and Cleaning**

Data monitoring and cleaning will be overseen by the data coordinating center (DCC) at the coordinating site. Data collection will be monitored on a weekly basis by the site study coordinator using the dashboard function on Survey solutions. The survey solution dashboard will consist of the following reports by study site: Date Household Census Completed, Number of Households Census Completed by Village, Percent Household Census Completed by village, Treatment Status by Worker, Age Distribution by Worker, Sex Distribution by Worker, GPS Missing by Worker, GPS Missing by Village, Number of Records Synced by Date, Assigned Treatment by Given Treatment, Treatment Status by Age, Treatment Status by Village, Age Distribution by Village, and Sex Distribution by Village.

The DCC will ensure that the site study coordinators log on to Survey solution weekly to confirm the status of the dashboard. In addition, upon each village census completion, the DCC will create and maintain a Stata program to identify data quality concerns. Any such concerns which must be addressed at the site specific level will be queried by the DCC. At every phase, as each village is completed and the data is considered cleaned, the data will be locked and a list of deaths will be generated and provided to each site for verbal autopsy.

# Appendix

Appendix 1. SAP

Appendix 2. Infant Adverse Events survey

Appendix 3. Pfizer Investigator Initiated Research Serious Adverse Event Report Form and Completion Guide

Appendix 4. Lab Protocol

Appendix 5. Community study forms

**References**

1 Porco TC, Gebre T, Ayele B, *et al.* Effect of Mass Distribution of Azithromycin for Trachoma Control on Overall Mortality in Ethiopian Children: A Randomized Trial. *JAMA* 2009; **302**: 962–8.

2 MORDOR Study Group. Mortality Reduction After Oral Azithromycin (MORDOR) Study. 2017.

3 Sie A, Louis VR, Gbangou A, *et al.* The Health and Demographic Surveillance System (HDSS) in Nouna, Burkina Faso, 1993–2007. *Global Health Action* 2010; **3**: 1–10.

4 Wang H, Bhutta ZA, Coates MM, *et al.* Global, regional, national, and selected subnational levels of stillbirths, neonatal, infant, and under-5 mortality, 1980–2015: a systematic analysis for the Global Burden of Disease Study 2015. *The Lancet* 2016; **388**: 1725–74.

5 Golding N, Burstein R, Longbottom J, *et al.* Mapping under-5 and neonatal mortality in Africa, 2000–15: a baseline analysis for the Sustainable Development Goals. *The Lancet* 2017; : 1–12.

6 Muller O, Garenne M, Kouyate B, Becher H. The association between protein–energy malnutrition, malaria morbidity and all-cause mortality in West African children. *Tropical Medicine & International Health* 2003; **8**: 507–11.

7 Becher H, Muller O, Dambach P, *et al.* Decreasing child mortality, spatial clustering and decreasing disparity in North-Western Burkina Faso. *Tropical Medicine & International Health* 2016; **21**: 546–55.

8 World Health Organization,, UNICEF. WHO/UNICEF Joint Statement: Managing possible serious bacterial infection in young infants 0–59 days old when referral is not feasible. 2017.

9 Yé M, Diboulo E, Niamba L, *et al.* An improved method for physician-certified verbal autopsy reduces the rate of discrepancy: experiences in the Nouna Health and Demographic Surveillance Site (NHDSS), Burkina Faso. *Population Health Metrics* 2011; **9**: 34.

10 Byass P, Herbst K, Fottrell E, *et al.* Comparing verbal autopsy cause of death findings as determined by physician coding and probabilistic modeling: a public health analysis of 54 000 deaths in Africa and Asia. *Journal of Global Health* 2015; **5**: 1–9.

11 Tobias J, Vutukuru S-R. Simple and rapid multiplex PCR for identification of the main human diarrheagenic Escherichia coli. *Microbiological Research* 2012; **167**: 564–70.
